# miR-181a is a novel player in the STAT3-mediated survival network of TCRαβ+ CD8+ T large granular lymphocyte leukemia

**DOI:** 10.1038/s41375-021-01480-2

**Published:** 2021-12-06

**Authors:** Jorn L. J. C. Assmann, Leticia G. Leon, Christiaan J. Stavast, Sanne E. van den Bogaerdt, Joyce Schilperoord-Vermeulen, Yorick Sandberg, Mar Bellido, Stefan J. Erkeland, David J. Feith, Thomas P. Loughran Jr, Anton W. Langerak

**Affiliations:** 1 Department of Immunology, Laboratory Medical Immunology, Erasmus MC, University Medical Center, Rotterdam, The Netherlands; 2 Department of Pathology, Netherlands Cancer Institute, Amsterdam, The Netherlands; 3 Department of Hematology, Maasstadziekenhuis, Rotterdam, The Netherlands; 4 Department of Hematology, Faculty of Medical Sciences, Groningen University Medical Center, Groningen, The Netherlands; 5 Division of Hematology/Oncology, Department of Medicine, UVA Cancer Center, University of Virginia, Charlottesville, VA USA; 6 ACE Rare Immunological Diseases Center, Erasmus MC, University Medical Center, Rotterdam, The Netherlands

**Keywords:** Chronic lymphocytic leukaemia, Oncogenesis

## Abstract

T-LGL cells arise as a consequence of chronic antigenic stimulation and inflammation and thrive because of constitutive activation of the STAT3 and ERK pathway. Notably, in 40% of patients, constitutive STAT3 activation is due to *STAT3* activating mutations, whereas in 60% this is unknown. As miRNAs are amongst the most potent regulators in health and disease, we hypothesized that aberrant miRNA expression could contribute to dysregulation of these pathways. miRNA sequencing in T-LGL leukemia cases and aged-matched healthy control TEMRA cells revealed overexpression of miR-181a. Furthermore, geneset enrichment analysis (GSEA) of downregulated targets of miR-181a implicated involvement in regulating STAT3 and ERK1/2 pathways. Flow cytometric analyses showed increased SOCS3+ and DUSP6+ T-LGL cells upon miR-181a inhibition. In addition, miR-181a-transfected human CD8+ T cells showed increased basal STAT3 and ERK1/2 phosphorylation. By using TL1, a human T-LGL cell line, we could show that miR-181a is an actor in T-LGL leukemia, driving STAT3 activation by SOCS3 inhibition and ERK1/2 phosphorylation by DUSP6 inhibition and verified this mechanism in an independent cell line. In addition, miR-181a inhibition resulted in a higher sensitivity to FAS-mediated apoptosis. Collectively, our data show that miR-181a could be the missing link to explain why *STAT3*-unmutated patients show hyperactive STAT3.

## Introduction

T-cell large granular lymphocyte (T-LGL) leukemia is a rare hematological neoplasia that is characterized by an abnormal expansion of T-LGL cells in the peripheral blood (PB) [1]. The precise disease etiology still remains elusive, although an initial persistent antigenic stimulation is thought to catalyze the proliferation of an (oligo)clonal population of T-LGL cells. In 2017, Lamy et al. proposed that the constitutive activation of signal transducer and activator of transcription 3 (STAT3) acts as a central hub in the proliferation and survival of the eventual clinically malignant monoclonal cell population. In addition, continuous activation of extracellular signal‑regulated protein kinase 1/2 (ERK1/2) seems to be involved in survival of T-LGL clones, albeit to a lesser extent [1, 2]. STAT3 has long been known for its fundamental pathogenic role in T-LGL leukemia because it acts on the expression of many genes related to cell survival. Specific inhibition of STAT3 with antisense oligos or through JAK inhibition with AG-490 in T-LGL leukemic cells restores their ability to go into apoptosis in vitro [3], clearly showcasing the importance of STAT3 in T-LGL survival. However, in only 30–40% of all patients constitutive STAT3 activation is the result of hot-spot mutations in the SH2 domain of *STAT3* [4], and so the reason why *STAT3* nonmutated patients still bear hyperactive STAT3 remains unsolved. Suppressor of cytokine signaling 3 (SOCS3) is one of the targets of the STAT3 pathway and is readily expressed upon STAT3 dimerization. SOCS3 forms an important negative feedback loop by suppressing JAK activity and is therefore also an important regulator of the STAT3 pathway [5]. Teramo *et al*. demonstrated that *SOCS3* mRNA expression is low in T-LGL cells although the gene was not epigenetically silenced and no clear mutation in the *SOCS3* gene was found [6].

MicroRNAs (miRNAs) are small noncoding RNAs of ~22 nucleotides with the ability to bind to 3′ untranslated regions (UTR) of messenger RNAs (mRNA). miRNA–mRNA interaction is orchestrated by Argonaute (AGO) proteins forming the miRNA-induced RISC complex, eventually leading to either mRNA degradation or translational disruption [7]. Of note, one miRNA can silence hundreds to thousands of genes and one gene can be regulated by multiple miRNAs [8, 9]. Site matching in the miRNA seed region occurs through 8mer, 7mer-m8, 7mer-A1, 6mer and offset 6mer matches [10], which is why 100% reverse complementarity to the seed region is not necessary to induce altered gene and therefore protein expression. miRNAs have been linked to various cellular processes, such as cell proliferation and apoptosis [11]. Also, miRNAs have been implicated in the leukemogenesis of both acute and chronic leukemias [12]. However, apart from a recent report on a miRNA-FasL axis that could possibly contribute to the development of neutropenia in T-LGL leukemia patients [13], a clear link between aberrant miRNA expression and T-LGL leukemia survival and proliferation has not been established [14, 15]. Therefore, we hypothesize here that miRNAs might be involved in the regulation of key molecules such as STAT3 and ERK1/2 in T-LGL leukemia.

In this study, we focused on deep sequencing of both miRNA and mRNA expression levels of T-LGL leukemia patients aiming to investigate the role of miRNAs in the aberrant proliferation capacity of these cells and their apoptosis resistance. We identified that the overexpression of one particular miRNA, miR-181a, which results in hyperactive STAT3 and ERK1/2 by decreasing SOCS3 and DUSP6 expression in T-LGL leukemia patient cells, eventually leads to resistance to FAS-mediated apoptosis. These findings corroborate the central role of STAT3 and ERK1/2 activation in T-LGL leukemia and provide an explanation for STAT3 hyper-activation in *STAT3* nonmutated patients.

## Materials and methods

### Patients, healthy controls and cell line

The biobank of the department of Immunology (Erasmus MC, University Medical Center, Rotterdam, The Netherlands) was retrospectively inspected in order to include persistent TCRαβ CD8 T-LGL proliferations in PB. Cases were included based on a combination of clinical, immunophenotypical, and molecular data (Supplementary Table 1). Use of T-LGL samples was approved by the Erasmus MC Institutional Review Board (MEC2015-617). Samples from all healthy blood donors were included upon informed consent and anonymized prior to use (MEC2016-202). A total of six TCRαβ+ CD8+ T-LGL leukemia cases and five age-matched healthy control samples were included for the miRNA-sequencing experiments, whereas an additional four TCRαβ+ CD8+ T-LGL leukemia cases and six healthy controls were included for RNA sequencing. Fifteen additional T-LGL leukemia cases were used as a validation cohort (MEC2018-0104). For in vitro miRNA inhibition assays, a human-derived T-LGL cell line (TL1) was used [16].

### Cell sorting

Patient cells were stained with fluorescent antibodies (Supplementary Table 2) and sorted based on aberrant expression levels of CD2, CD5, and/or CD7 and/or high expression of CD57. Age-matched healthy control PBMCs were stained with fluorescent antibodies (Supplementary Table 2) and terminally differentiated effector memory cells (CD3+/CD8+/TCRγδ−/CD45RA+/CCR7−; TEMRA) were sorted as control populations. All sorting experiments were performed using the FACS Aria III instrument (BD Biosciences, San Jose, CA, USA).

After transfection, cells were stained with fluorescent antibodies (Supplementary Table 2) and GFP-positive and -negative fractions of CD8+ T cells were sorted (CD3+/CD8+/TCRγδ−).

### RNA isolation

Cells were lysed in RLT buffer+ (Qiagen, Hilden, Germany) containing 1% β-mercapto-ethanol or into TRIzol (Invitrogen, Carlsbad, USA) after which combined DNA/RNA (Qiagen DNA/RNA/miRNA Allprep kit) or RNA was isolated according to the manufacturer’s protocol. RNA integrity was checked on the Agilent 4200 TapeStation system (Agilent, Santa Clara, CA).

### miRNA and RNA sequencing

miRNA libraries were prepared using the CleanTag™ Small RNA Library Prep Kit (Cat# L-3206) (Trilink Biotechnologies, San Diego, CA) according to the manufacturer’s protocol. cDNA libraries were individually quantified on a high sensitivity DNA chip. Next, libraries were pooled equimolarly and sequenced on the Illumina HiSeq 2000 platform (Illumina, San Diego, CA) at the department of Hematology (Erasmus MC). Data can be found under accession number GSE159878.

For RNA sequencing, 1 ng of total RNA was used as input material and libraries were prepared following the SMARTer-seq v4 Ultra low input RNA kit for sequencing (Takara Bio USA, Mountain View, CA, USA). Sequencing was performed on the Illumina NovaSeq 6000 platform (Illumina, San Diego, CA). Data can be found under accession number GSE159819.

### Real-time quantitative PCR

Experiments were performed using TaqMan miRNA assays (Thermo Fisher, Waltham, MA, USA Cat: 4427975; ID 00480), universal PCR mastermix (Applied Biosystems) and run on a Quantstudio 3 (Thermo Fisher, Waltham, MA, USA). C_t_ values were normalized against the snRNA U6 (Thermo Fisher, Cat: 4427975; ID:001973).

### Bioinformatic analyses of miRNA and RNA-sequencing data

After the sequencing and demultiplexing procedure, sequencing quality was determined with FastQC (v 0.11.8). Next, fastq files were aligned with STAR aligner (v.2.5.3e) for the RNAseq data, using GRCh38 as reference. The miRNA data were trimmed and aligned using Novoalign. Counts data were obtained via FeatureCounts (v1.6.0) using Homo_sapiens.GRCh38.92 as reference for the RNAseq data and miRBase.annotation20 for the miRNA data. Before starting downstream analysis, alignment and assigned reads quality were determined with MultiQC, during which some of the samples had to be discarded, as they did not pass the quality threshold. Noise reduction was applied to the count data removing the very low counts reads, then rlog transformation was used for visualization and comparison purposes, which included correlation and clustering analysis to generate heatmaps and PCA plots. For differential expression analysis, DESeq2 (DESeq2 R package (v1.22.2) was used.

### Transfection and modulation of miR181

Overall, 10 × 10^6^ total PBMCs or cells of the TL1 cell line were transfected with 1 μg miR-181ab pLX301 vector (full data on vector construction available upon request from corresponding author) or 4 μg empty vector (EV) control using the Amaxa Nucleofector II system and Amaxa human T cell nucleofector kit (Lonza, Gaithersburg, MD, USA) at program U-14 according to the manufacturer’s protocol.

For inhibitor experiments in primary T-LGL samples, 25 nM of miR-181a power inhibitor (miRCURY) or inhibitor scrambled control was used to assess SOCS3 and DUSP6 levels. Cells were not transfected, the inhibitor was delivered through gymnosis [17].

For miR-181a inhibitor studies in the TL1 cell line, 25 nM of miR-181a inhibitor (miRCURY) or inhibitor scrambled control was used to assess pSTAT3 and pERK1/2 levels. Alternatively, for analysis of SOCS3 and DUSP6 protein levels a cotransfection was performed using an empty mCherry expressing vector together with the LNA miRNA inhibitor or control in a 1:4 ratio, followed by gating on mCherry positive cells. In the experiment aiming at analysis of SOCS3 and DUSP6 protein levels, cells were starved from IL2 overnight prior to measuring protein levels.

For miR-181a overexpression experiments in HeLa cells, 150 pmol of miR-181a mimic (miRCURY) was transfected using lipofectamine2000 reagent (Promega) according to the manufacturer’s protocol. Forty-eight hours post transfection SOCS3, DUSP6, pSTAT3 and pERK1/2 levels were determined by western blot.

In all experiments basal expression levels were investigated, i.e., no extra stimuli were added.

### Phospho-flow analysis

T-LGL cells were stained 24 h post inhibitor treatment using the PERFIX EXPOSE kit (Beckman Coulter) according to the manufacturer’s protocol. Cells were either stained with DUSP6 antibody or with SOCS3 primary antibody followed by a secondary fluorescent antibody (Supplementary Table 2).

All other cells were stained 48 h post transfection using the PERFIX EXPOSE kit (Beckman Coulter) according to the manufacturer’s protocol. Cells were either stained with BD Phosflow antibodies or with a primary antibody followed by a secondary fluorescent antibody (Supplementary Table 2). Samples were analyzed on the FACS LSR Fortessa flow cytometer (BD Biosciences).

### Apoptosis assay and cell cycle analysis

Cells of the TL1 cell line were transfected with LNA miR-181a inhibitor (25 nM) or scrambled control (25 nM). Forty-eight hours post transfection the cells were seeded in a 96-wells plate at a concentration of 2.5 × 10^6^/ml and stimulated overnight with the agonistic CD95 antibody (0.5 mg/ml, Biolegend). The % increase in apoptotic cells was calculated as the % apoptotic cells in the stimulated condition − % apoptotic cells in the unstimulated condition.

Cell cycle analysis was performed as previously described [18].

### Dual-luciferase reporter assay and vector

HEK293 cells were plated at 25% confluence 24 h prior to transfection. A cotransfection of a pmirGLO Dual-Luciferase miRNA target expression vector (pmirGLO) (Promega, USA) with *SOCS3* or *DUSP6* WT 3′ UTR or MT 3′ UTR (Supplementary Table 3), together with LNA-hsa-miRNA-181a (miRCURY) was performed in a 1:6 ratio with FuGENE6 (Promega, USA) according to the manufacturer’s protocol. Twenty-forty hours post transfection luciferase and Renilla luminescence were measured sequentially on a flow cytometric Discover microplate reader (Promega, USA).

### Statistical analysis

Mann–Whitney *U* test or Student’s *t* test were used to test differences between groups. Two-sided *P* values of less than 0.05 were considered statistically significant. Statistical tests and levels of significance are indicated in the figure legends.

## Results

### miR-181a is upregulated in all T-LGL cases compared to healthy control TEMRA T cells

To gain insight into the miRNA profile of T-LGL leukemia cells in view of its possible contribution to disease pathogenesis, we deep sequenced miRNAs from purified cells of a total of six TCRαβ+ CD8+ T-LGL patients and five sorted age-matched healthy control CD8+ TEMRA cell fractions as the closest normal counterpart cell, using a next-generation sequencing approach. Characteristics of all patients that were included in this study are shown in Supplementary Table 1.

**Fig. 1:**
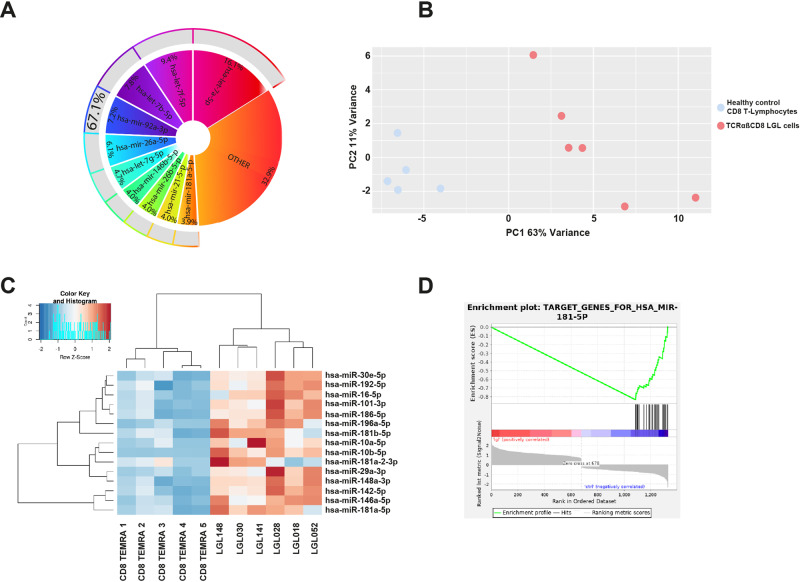
Characterization of the T-LGL microRNome. Top ten overexpressed microRNAs between TCRαβ+ CD8+ T-LGL cells and healthy control TEMRA cells (**A**). PCA analysis of TCRαβ+ CD8+ T-LGL cells compared to healthy control TEMRA cells (**B**). Heatmap displaying the top 20 differentially expressed miRNAs between TCRαβ+ CD8+ T-LGL cells and healthy control TEMRA cells (**C**). Geneset enrichment analysis of miRNA181-5p correlating downregulated mRNA targets [40] with expression of miRNA181-5p based on seed regions (**D**).

Unsupervised analysis indicated a total of 102 differentially expressed miRNAs with an adjusted *P* value of less than 0.05 and a log2 fold change (FC) of higher than 1 or lower than −1 (Supplementary Fig. 1). A total of 94 miRNAs appeared to be upregulated in T-LGL compared to normal, whereas only 8 were downregulated (Supplementary Table 4). The top ten overexpressed miRNAs accounted for 67% of the normalized reads, with the relative abundance being expressed as the percentage of total normalized mature miRNA reads (Fig. 1A). Principal component analysis (PCA) revealed that T-LGL leukemia patient cells and healthy control TEMRA cells could be clearly distinguished by differential miRNA expression (Fig. 1B).

Because PCA analysis displayed well-defined clusters of T-LGL cells and control CD8+ TEMRA cells, further in depth analysis was focused on those miRNAs that were upregulated in all T-LGL leukemia cell samples compared to healthy control cells based on unsupervised hierarchical clustering analysis (Fig. 1C). By integration of miRNA-sequencing and RNA-sequencing datasets through geneset enrichment analysis (GSEA) on four selected miRNAs that were upregulated in all T-LGL patient samples compared to control CD8+ TEMRA cells, we aimed to explore their involvement in T-LGL disease pathogenesis. During this approach we primarily focused on potential mRNA targets that were downregulated in all T-LGL cases as compared to controls and selected on mRNA targets that could be of relevance in T-LGL disease pathology specifically (Fig. 1D). This analysis revealed a list of mRNA targets for miR-181a, with *SOCS3* and *DUSP6* mRNAs as most intriguing targets (Supplementary Fig. 2). Differential expression of all miR-181a targets can be found in Supplementary Table 5. We then determined expression of miR-181a in a second cohort of 15 TCRαβ+ CD8+ T-LGL patients by qPCR and found it to be upregulated in virtually all cases compared to healthy control TEMRA cells, thus validating the elevated expression of miR-181a of our NGS cohort (average 4.7 range: 1.4–11.8; Supplementary Fig. 3). *STAT3* mutated patients are depicted in bold.

### Blocking of miR-181a in patient T-LGL cells results in SOCS3 and DUSP6 upregulation

**Fig. 2:**
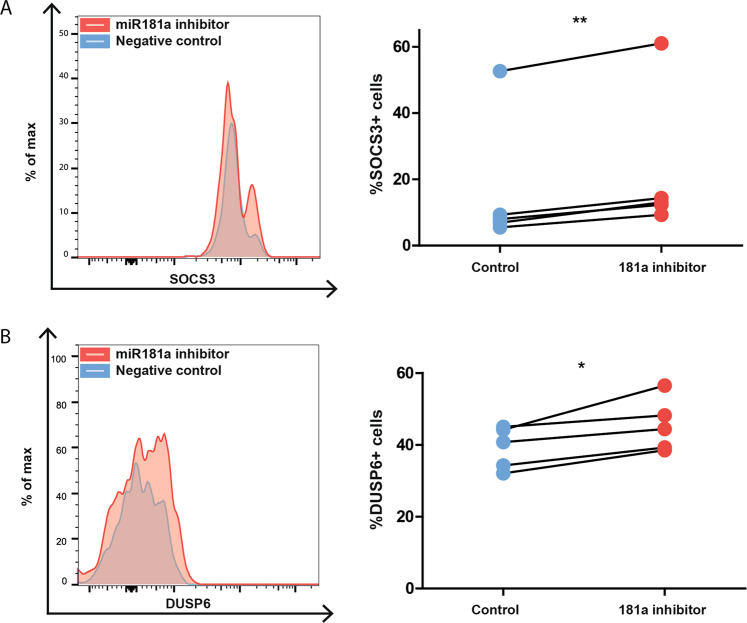
SOCS3 and DUSP6 increase upon miR-181a inhibition in primary T-LGL cells. Representative FACS plot (left) and %SOCS3+ T-LGL cells 24 h post treatment (*n* = 5) (right) (**A**). Representative FACS plot (left) and %DUSP6+ T-LGL cells 24 h post treatment (*n* = 5) (right) (**B**). Graphs indicate mean plus SD. Gating strategy is depicted in Supplementary Fig. 9. Statistical significance was tested using the paired *T*-test. Levels of significance indicated in the plots: *p < 0.05, **p < 0.01. As a negative control, a control miRNA mimic was delivered into the cell through gymnosis.

The putative miR-181a-mediated repression of *SOCS3* and *DUSP6* mRNA was striking, since these are crucial negative regulators of JAK/STAT and ERK pathways, respectively [19, 20]. Both pathways are known to be dysregulated in T-LGL leukemia and to mediate cell survival of T-LGL leukemia cells through generating pro-survival signals and mediating resistance to FAS induced apoptosis [21]. Following inhibition of miR-181a, the percentage of basal SOCS3+ cells in all primary T-LGL samples tested increased (average 5.5%; Fig. 2A, *p* = 0.003), whereas this effect was absent in healthy CD8 T cells within the same patient (Supplementary Fig. 4A). Of note, the percentage of SOCS3+ cells in healthy CD8 T cells was much higher than in T-LGL cells. The percentage of basal DUSP6+ cells on in T-LGL cell samples increased (average 6.1%; Fig. 2B, *p* = 0.02), while in healthy CD8 T cells within the same patient this effect was not observed (Supplementary Fig. 4B). Of note, miR-181a upregulation in primary T-LGL cells over control CD8 TEMRA cells ranged from 1.5 to 11.5 times, whereas the fold increase was only 0.2–1.6 for miR-181b (data not shown).

### Overexpression of miR-181a in control CD8+ T cells results in STAT3 and ERK1/2 phosphorylation

To further study the role of miR-181a in T-LGL leukemia pathogenesis, we mimicked the effect of miR-181a overexpression by transfecting primary healthy age-matched PBMCs with the miR-181ab pLX301 vector (Supplementary Table 6).

Following transfection, healthy control CD8+ T cells were sorted and subjected to qPCR analysis to evaluate miR-181a expression. Indeed, 48 h post transfection we observed a relative increase in miR-181a expression of 7.31 (1.6–24.1) in CD3+CD8+GFP+ T cells over CD3+CD8+GFP− T cells (Supplementary Fig. 5).

**Fig. 3:**
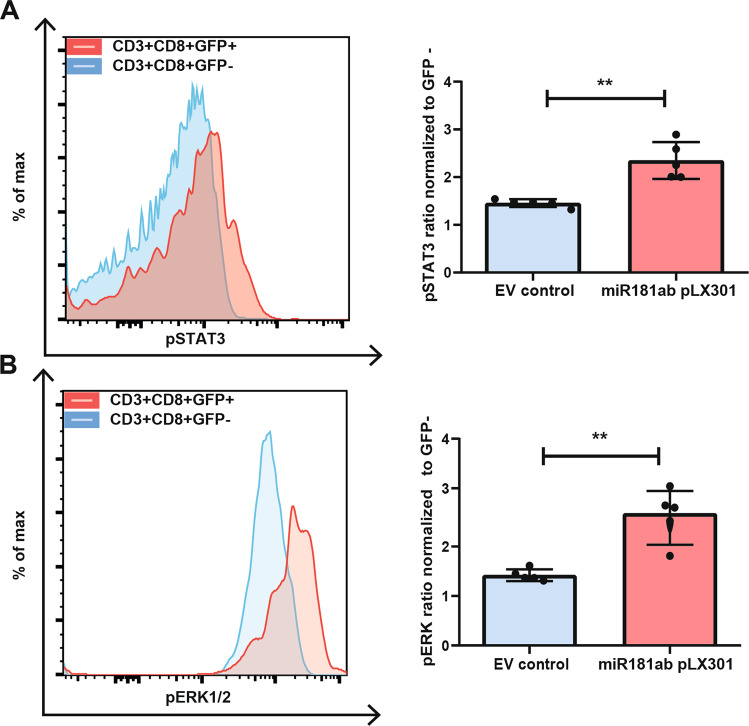
STAT3 and ERK1/2 phosphorylation increase with high miR-181a expression in healthy control CD8+ T cells. Representative FACS plot (left) and pSTAT3 MFI ratio normalized to GFP− CD8+ T cells 48 h post transfection (*n* = 5) (right) (**A**). Representative FACS plot (left) and pERK1/2 MFI ratio normalized to GFP− CD8+ T cells 48 h post transfection (*n* = 5) (right) (**B**). Graphs indicate mean plus SD. Gating strategy is depicted in Supplementary Fig. 9b. Statistical significance was tested using the Mann–Whitney *U* test. Levels of significance indicated in the plots: *p < 0.05, **p < 0.01. As a negative control an empty GFP expressing vector was transfected into the cell.

Transfection of healthy PBMCs with the miR-181ab pLX301 vector resulted in an increase of the basal pSTAT3 MFI ratio to 2.47 (2.00–2.89) (*p* = 0.008) in the CD3+CD8+GFP+ cells over the GFP− fraction 48 h post transfection, whereas the CD3+CD8+ population following EV control transfection remained largely unaffected (Fig. 3A).

Regarding pERK1/2 expression, 48 h post transfection of healthy PBMCs with the miR-181ab pLX301 vector also an increase of the basal pERK1/2 MFI ratio to 2.49 (1.81–3.04) (*p* = 0.008) was detected in the CD3+CD8+GFP+ subpopulation over the GFP− population, while the CD3+CD8+ population within the EV control remained unaffected (Fig. 3B).

### Inhibition of miR-181a in TL1 cells results in STAT3 and ERK1/2 de-phosphorylation

**Fig. 4:**
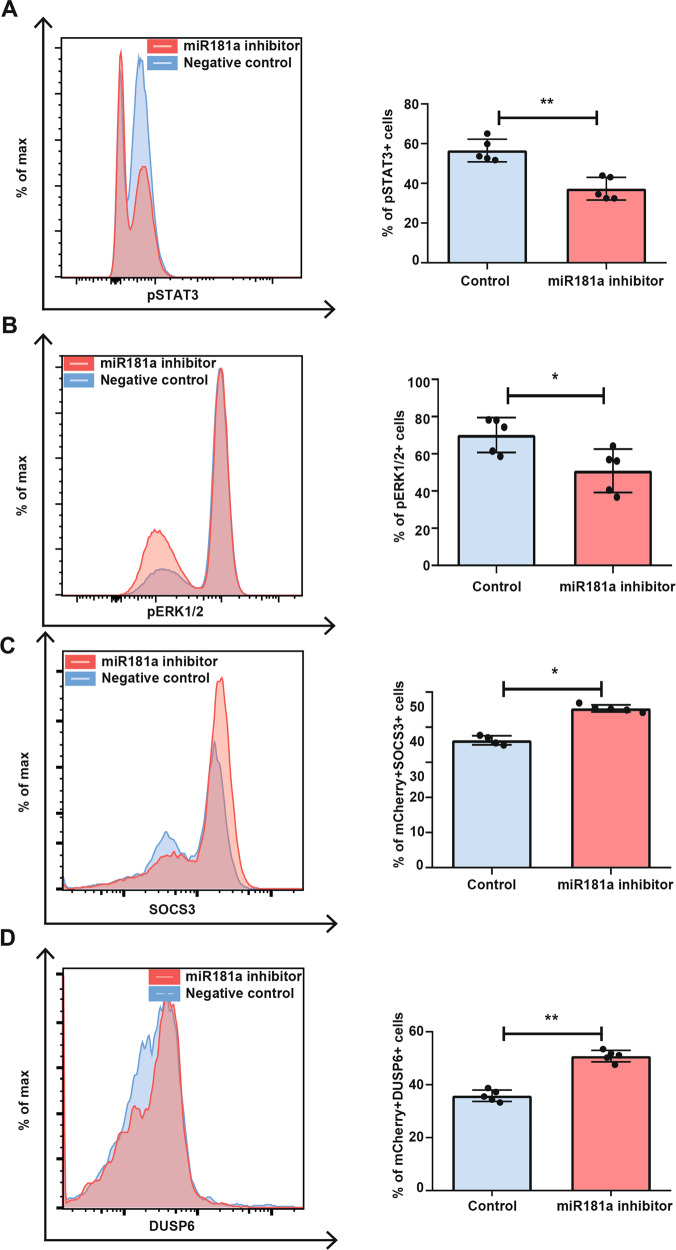
STAT3 and ERK1/2 phosphorylation is decreased in a SOCS3 and DUSP6-dependent manner by blocking miR-181a expression in TL1 cells. Representative FACS plot (left) and %pSTAT3+ TL1 cells 48 h post transfection (*n* = 5) (right) (**A**). Representative FACS plot (left) and %pERK1/2+ TL1 cells 48 h post transfection (*n* = 5) (right) (**B**). Representative FACS plot (left) and %SOCS3+ TL1 cells 48 h post transfection (*n* = 5) (*n* = 4 controls) (right) (**C**). Representative FACS plot (left) and %DUSP6+ TL1 cells 48 h post transfection (*n* = 5) (right) (**D**). Graphs indicate mean plus SD. Gating strategy is depicted in Supplementary Fig. 9c. Statistical significance was tested using the Mann–Whitney *U* test. Levels of significance indicated in the plots: *p < 0.05, **p < 0.01. As a negative control, a mix of nonspecific miRNA inhibitors was transfected into the cell.

To further validate the involvement of miR-181a in T-LGL leukemia, we made use of the T-LGL model cell line (TL1) for a miR-181a expression inhibition experiment with the LNA miRNA-181a inhibitor (miRCURY). Expression miR-181a was found to be high in this cell line and was expressed 11.5 times more than the average of healthy control TEMRA CD8+ T cells. Keeping in mind that SOCS3 and DUSP6 were found as primary targets, we focused on the STAT3 and ERK1/2 pathways, also because these are postulated to be hyper-activated in T-LGL primary cells. To this end, basal pSTAT3 levels were evaluated 48 h post transfection, showing a decrease of 19.5% (18.1–21.2) in pSTAT3+ cells compared with the scrambled control (*p* = 0.008) (Fig. 4A). Of note, total STAT3 levels were unaffected (Supplementary Fig. 6C). In addition, the effect of miR-181a downregulation on ERK1/2 in TL1 cells was investigated, resulting in a 17.8% (10.2–22.1) decrease basal in pERK1/2 positive cells compared with the scrambled control (*p* = 0.0317) (Fig. 4B). Total ERK1/2 levels were unaffected (Supplementary Fig. 6D).

### The miR-181a regulatory effect in TL1 cells is mediated through increased SOCS3 and DUSP6 expression

To fully confirm the mechanism of action of miR-181a in regulating the STAT3 and ERK1/2 pathways, we then performed flow cytometric analyses focusing on the predicted targets SOCS3 and DUSP6. To this end, we cotransfected mCherry expressing vector together with a miR-181a inhibitor or scrambled control into TL1 cells. SOCS3 and DUSP6 protein expression was assessed in the mCherry positive cell fraction 48 h post transfection. Upon inhibition of miR-181a, we observed an average increase of 9.0% in basal SOCS3 expressing cells compared to the scrambled control (Fig. 4C) (*p* = 0.016), whereas a 15.6% increase in basal DUSP6 expressing cells was seen upon blocking miR-181a 48 h post transfection (Fig. 4D) (*p* = 0.008). In addition, western blot analysis showed an increased FC of SOCS3 and DUSP6 protein after miR-181a inhibition compared to negative control that trended towards significance (Supplementary Fig. 6A, B).

### SOCS3 and DUSP6 3′ UTRs are targeted by miR-181a

**Fig. 5:**
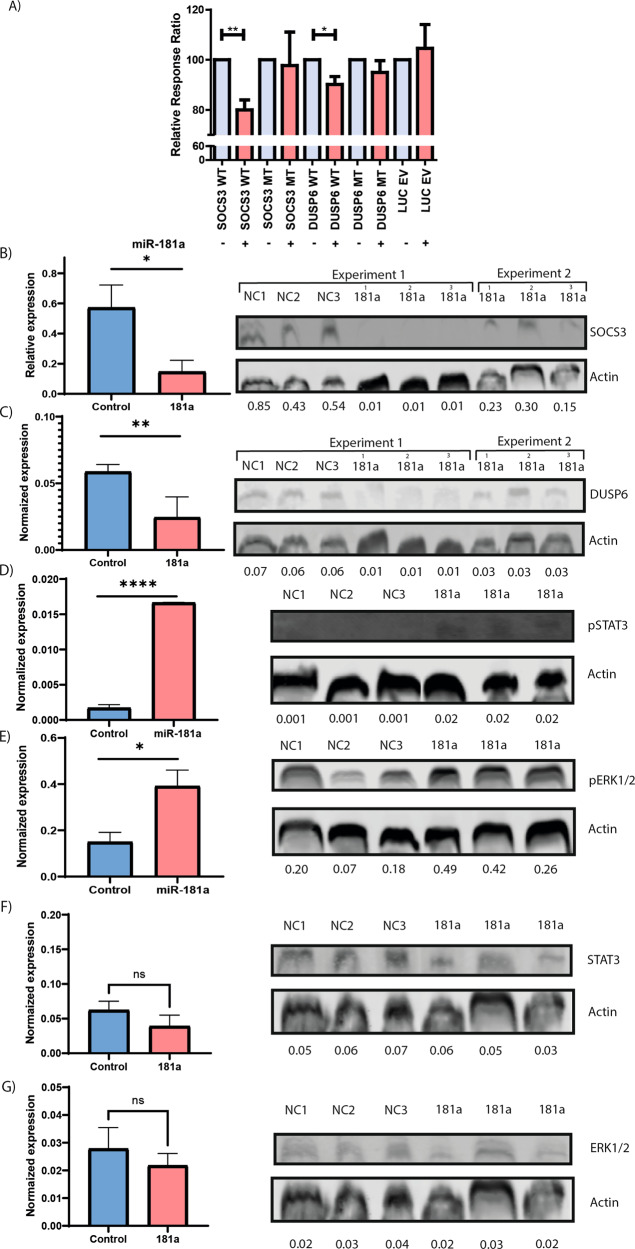
SOCS3 and DUSP6 3′ UTRs are directly bound by miR-181a. Luciferase constructs were cotransfected into HEK293 cells together with miR-181a. Luciferase activity was determined 24 h after transfection. The ratio of normalized inhibitor to control luciferase activity is shown. Data represent two independent experiments with three measurements. Graphs indicate mean plus SEM (**A**). A miR-181a mimic or scrambled control was transfected into HeLa cells and SOCS3 (**B**), DUSP6 (**C**), pSTAT3 (**D**), pERK1/2 (**E**), STAT3 (**F**) and ERK1/2 (**G**) protein levels were determined by western blot 48 h post transfection. Statistical significance was tested with the paired Student’s *t* test (**A**) or normal Student’s *t* test (**B**–**G**) with single measurements. Levels of significance indicated in the plots: *p < 0.05, **p < 0.01, ***p < 0.001. Of note, membranes of western blots under **D**–**G** are cut between lanes. All samples were run on the same blot and same membrane and were measured all together in one single measurement.

To assess the direct interaction of miR-181a with *SOCS3* and *DUSP6* 3′ UTRs we cotransfected a LNA miRNA-181a mimic together with wild-type (WT) or mutant (M) 3′ UTRs of *SOCS3* and *DUSP6*. Indeed, cotransfection lead to a 20.0 and 9.8% decrease of the relative luminescence in *SOCS3* and *DUSP6* WT 3′ UTRs, whereas the corresponding M 3′ UTRs did not show any differences in luminescence (Fig. 5A), thus confirming direct binding of miR-181a.

### pSTAT3 and pERK1/2 increase in a SOCS3 and DUSP6-dependent manner upon transfection of a miR-181a mimic

To prove that miR-181a is directly involved in pSTAT3 and pERK1/2 phosphorylation through regulation of SOCS3 and DUSP6 levels, we transfected a miR-181a mimic in HeLa cells, which are known to have a high transfection efficiency (>95%). We observed that miR-181a upregulation decreases basal SOCS3 (*p* < 0.05) and DUSP6 (*p* < 0.01) expression on western blot (Fig. 5B, C), while concomitantly basal pSTAT3 (*p* < 0.001) and pERK1/2 (*p* < 0.05) levels were increased without affecting total STAT3 and ERK1/2 levels (Fig. 5D–G). These data thus clearly depict miR-181a-mediated protein repression of SOCS3 and DUSP6 leading to pSTAT3 and pERK1/2 phosphorylation.

### miR-181a inhibition in TL1 cells results in higher sensitivity to FAS-mediated apoptosis, but does not affect the cell cycle

**Fig. 6:**
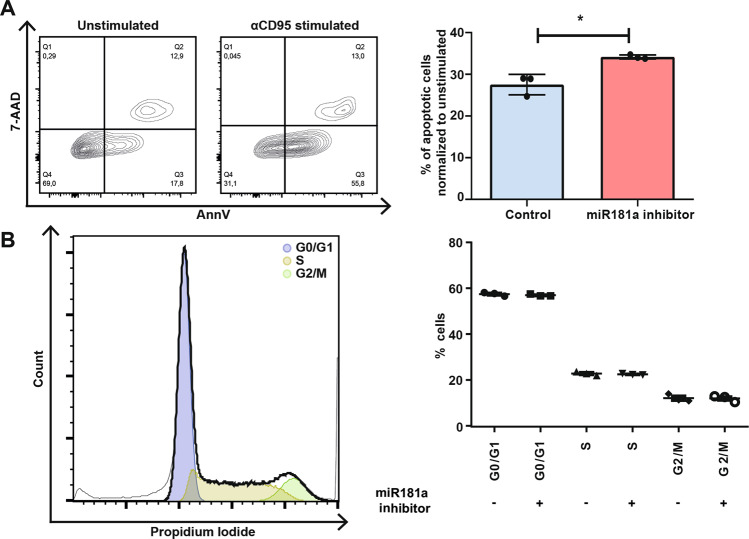
Blocking of miR-181a in TL1 cells results in higher sensitivity to FAS-mediated apoptosis, without altering the cell cycle. Representative FACS plot (left) and % of apoptotic cells normalized to unstimulated 48 h post transfection and overnight stimulation with the agonistic CD95 antibody (*n* = 3) (right) (**A**). Representative FACS plot (left) and % cells in sub G1, G0/G1, S phase or G2/M phase (*n* = 3) (**B**). Graphs indicate mean plus SD. Statistical significance was tested using the Student’s *t* test. Levels of significance indicated in the plots: *p < 0.05. As a negative control, a mix of nonspecific miRNA inhibitors was transfected into the cell.

Since ERK1/2 and especially STAT3 are known to be involved in survival of T-LGL leukemia cells through inhibition of FAS-mediated apoptosis, TL1 cells were transfected with 25 nM LNA miR-181a inhibitor and 48 h post transfection cells were incubated overnight with the agonistic CD95 antibody to evaluate apoptosis. In the miR-181a-inhibited group higher frequencies of apoptotic cells were observed after CD95 stimulation, with an average percentage of 34.3% (33.8–34.7) of apoptotic cells after normalization to the unstimulated condition, contrary to the mock control that displayed an average of 27.5% (24.7–29.0) of apoptotic cells after normalization (*p* = 0.01) (Fig. 6A). In contrast, cell cycle analysis of TL1 cells did not show any differences in G0/G1, G2/S fractions of the cells between both conditions (Fig. 6B).

## Discussion

STAT3 is generally believed to act centrally in the survival of TCRαβ CD8 T-LGL leukemia cells [2]. Since the discovery of *STAT3* SH2 domain hot-spot mutations in T-LGL leukemia cells, STAT3 hyper-activation could be explained in 30–40% of cases [22]. Nonetheless a large subset of T-LGL patients still bears cells with hyperactive STAT3, without any discernible genetic aberrancies. As miRNAs are one of the most potent epigenetic regulators in both health and disease [23], we hypothesized a role for miRNAs in STAT3 hyper-activation, and thus generated the first high- throughput NGS miRNA dataset in T-LGL leukemia. Both unsupervised hierarchical clustering and PCA analyses revealed that T-LGL cells could be separated from their healthy, age-matched control CD8+ TEMRA subsets based on mature miRNA expression. The separation of T-LGL cells from their healthy counterparts suggested that one or more miRNAs could be potentially directly involved in cell survival and/or proliferation of T-LGL cells. Therefore, we deep sequenced the mRNA transcripts of T-LGL patients and healthy control CD8+ TEMRA cells and cross-analyzed both datasets in order to correlate high expression of selected miRNAs to low expression of their candidate mRNA targets. GSEA analysis revealed a strong inverse correlation between miR-181a expression and *SOCS3* and *DUSP6* mRNA expression, known to be involved in the regulation of the STAT3 and ERK1/2 signaling pathways, respectively. The precise etiology behind miR-181a overexpression remains elusive, as it can be transcribed from two different genes (*MIR181A1* and *MIR181A2*) which can be regulated by a 683 predicted transcription factors that can bind to eight known enhancer and two known promoter/enhancer regions and are an interesting target for future studies.

SOCS3 is the main regulator of the STAT3 pathway and exerts its effect by binding to JAK via its N-terminal kinase inhibitory region (KIR), thus directly interfering with JAK’s catalytic activity [24]. Furthermore, SOCS3 directly binds to the cytokine receptor, thereby gaining specificity since it is only bound firmly to JAK when the kinase is in close proximity to the cytokine receptor [25, 26]. SOCS3 proteins are readily synthesized after STAT3 phosphorylation induced by IL6, but also by IL2, thus inhibiting the STAT3 cascade through a classical negative feedback loop [5, 27]. Our RNA-sequencing dataset indicated that *SOCS3* expression is low in T-LGL cells compared to healthy control CD8 TEMRA cells. Even though this might seem counter-intuitive given that in other cell contexts *SOCS3* expression is induced by pSTAT3 activation, this is in line with earlier literature on *SOCS3* expression in T-LGL leukemia [6]. Our results highlight the increase of pSTAT3 expression upon overexpression of miR-181a in CD8+ T cells, which is corroborated by the observation that pSTAT3 was decreased in an SOCS3-dependent manner upon blocking miR-181a in a T-LGL cell line model. Furthermore, pSTAT3 increased in a SOCS3-dependent manner upon transfection of a miR-181a mimic in the HeLa cell line and SOCS3 expression increased in T-LGL patient cells upon miR-181a inhibition. Lastly, cotransfection of a miR-181a mimic with *SOCS3* 3′ UTR resulted in a decreased relative luminescence, providing evidence for the direct binding of miR-181a to the *SOCS3* transcript. Our findings might thus provide the missing link as to why SOCS3 mRNA and protein expression are low in T-LGL leukemia, as indicated by Teramo et al. in 2013 [6]Furthermore, these findings also reveal why *STAT3* nonmutated patients also bear hyper-activation of the STAT3 molecule. miR-181a expression did not seem to be influenced by mutations in the *STAT3* gene as four of our T-LGL patients in the combined cohorts showed *STAT3* mutations, yet still had high expression of miR-181a. It might be possible that miR-148-3p and the miR-221/222 cluster have an additional effect on SOCS3 repression, since these might also target the *SOCS3* 3′ UTR and are moderately expressed in T-LGL leukemia cells.

Although less extensively described, the ERK1/2 pathway is constitutively activated in T-LGL leukemia as well [21]. ERK1/2 is part of the Ras/Raf/MEK/ERK pathway and is amongst others activated through cytokine receptor signaling [28]. Regulation of the ERK1/2 pathway is performed at many levels by scaffold proteins and phosphatases of which the dual specificity phosphatase (DUSP) family it most known for its important regulatory function [29]. Dysregulation of the ERK1/2 pathway usually leads to aberrant proliferation and apoptosis resistance [30]. DUSP6 is specific for ERK1/2 and has been previously described as a regulatory molecule in T cells [31]. Moreover, it has been validated as target of miR-181a in mouse T cells [32]. Our results underline a role for miR-181a in DUSP6 regulation, since (1) miR-181a inhibition in primary T-LGL cells resulted in an increase of DUSP6+ cells, (2) inhibition of miR-181a in the TL1 T-LGL cell line model resulted in an increase in DUSP6+ cells and (3) cotransfection of a miR-181a mimic with the *DUSP6* 3′ UTR resulted in a decreased relative luminescence. Also, pERK1/2 increased in a DUSP6-dependent manner upon transfection of a miR-181a mimic in the HeLa cell line. Together, this clearly indicates that miR-181a can also induce ERK1/2 phosphorylation by interfering with DUSP6 expression. Of note, miR25-3p could potentially exert an extra effect on DUSP6 downregulation.

From the 1371 predicted targets of miR-181a, Li et al. validated PTPN22 and SHP2 in a murine model. Both are phosphatases that can potentially negatively regulate tyrosine kinase activity of ERK1/2 and STAT3 [32]. PTPN22 exerts its effect downstream of the TCR, where it can dephosphorylate LCK and ZAP70 [33]. As we did not crosslink the TCR in our experiments, this basically rules out an effect of PTPN22 on ERK1/2 phosphorylation in T-LGL cells. SHP2 phosphatase has been reported to have dual and opposite roles in STAT3 phosphorylation [34–36]. Although we cannot completely rule out an effect of SHP2 on STAT3 phosphorylation, our RNA-sequencing data showed no differential regulation of the *PTPN11* gene that encodes the SHP2 phosphatase, thus making a role for SHP2 in T-LGL cells less likely. Also, western blot analysis of SHP2 protein after transfection of a miR-181a inhibitor in the TL1 cell line model did not show any differences (Supplementary Fig. 7).

In theory, miR-181b and miR-181c, which are also upregulated in the RNA-sequencing dataset, could also have regulatory effects on SOCS3 and DUSP6. Since miR-181c expression was low across all samples we speculate that this miRNA would not exert any effect. Since miR-181b was also upregulated according to our RNA-sequencing dataset, we measured its expression in an additional cohort of 15 T-LGL patients and 6 healthy controls and found no significant difference, thus ruling out the possibility that miR-181b would have an extra regulatory effect in T-LGL leukemia (Supplementary Fig. 8).

Both the STAT3 and ERK1/2 pathway are involved in cell proliferation, differentiation and apoptosis resistance in many different cell types, and are particularly involved in apoptosis resistance of T-LGL cells [3, 37]. Central in T-LGL leukemia is the FAS pathway, since T-LGL cells are known to be resistant to FAS-mediated apoptosis [38]. After miR-181a inhibition, TL1 LGL cells displayed re-sensitization to FAS-mediated apoptosis in an apoptosis assay. In contrast, TL1 LGL cells did not display differences in cell cycle upon miR-181a inhibition. These results are in line with current literature, as T-LGL proliferations are not thought to be the result of rapidly proliferating cells, but more so of dormant cells that refrain from going into apoptosis [39].

Collectively, our results underline the idea that targeted therapy of the STAT3 pathway for T-LGL patients in the clinic would be beneficial. First line therapy for T-LGL leukemia patients mostly still relies on single immunosuppressive agents that are orally ingested. Usually, methotrexate, cyclophosphamide or cyclosporine A is chosen as therapeutic option and patients have to be on treatment for at least 4 months before assessing response. Overall response rates do not exceed 30% and, as these drugs suppress the immune system as a whole, increasing infection rates are observed as an adverse side effect in these patients [30]. Furthermore, patients eventually need combination therapy to achieve a complete response. Tofacitinib citrate is a JAK inhibitor that has FDA approval for rheumatoid arthritis. Bilori *et al*. suggested tofacitinib as a novel therapy for refractory T-LGL leukemia and in their cohort of nine patients that were refractory to first and second line therapy, 66% of the patients achieved complete hematological remission. The use of tofacitinib was without any severe adverse effects and, even though other clinical trials with tofacitinib exhibited that patients with normal hematopoiesis could eventually display neutropenia or anemia, patients with T-LGL leukemia clearly show hematological improvement [31]. Even though current first line therapy and the use of tofacitinib are applicable in T-LGL leukemia, directly inhibiting miR-181a with an LNA inhibitor as a therapeutic strategy might also be an interesting option. Miravirsen (SPC3649), is an LNA inhibitor of miR-122, that is in phase II clinical trials for treatment of hepatitis C virus and shows great potential [32]. While the recent progression of LNA inhibitors to phase II clinical trials is promising, the lack of more efficient and specific delivery methods remains a major barrier in the development of miRNA-based therapies. Even though the rationale and potential for LNA inhibitors in therapy of T-LGL leukemia is there, it remains to be seen if and when these will indeed reach the clinic.

Taken together, in T-LGL leukemia miR-181a overexpression results in dysregulation of two key pathways. The ERK pathway is dysregulated trough inhibition of the DUSP6 protein, which is the direct cytoplasmic inhibitor of phosphorylated ERK1/2. Notably, the STAT3 pathway is dysregulated as well, in particular through the inhibition of SOCS3, a protein that is absent in all T-LGL leukemia cells. The finding of an miR-181a-SOCS3-pSTAT3 axis in T-LGL leukemia is of broader importance, as it could potentially clarify why *STAT3* nonmutated patients also acquire hyper-activation of the STAT3 molecule. These findings further strengthen the idea that STAT3 plays a central role in T-LGL leukemia, with miR181 being a new player in the dysregulation of STAT3.

## Supplementary information

Supplementary figures

Supplementary tables
