# Supplementary material for: miR-181a is a novel player in the STAT3-mediated survival network of TCRαβ+ CD8+ T large granular lymphocyte leukemia
Source: Leukemia. 2021 Dec 6;36(4):983–93. doi: 10.1038/s41375-021-01480-2 (PMC8979821; doi:10.1038/s41375-021-01480-2)
Supplement: Supplementary file 1 — Supplementary figures [file 41375_2021_1480_MOESM1_ESM.pdf]

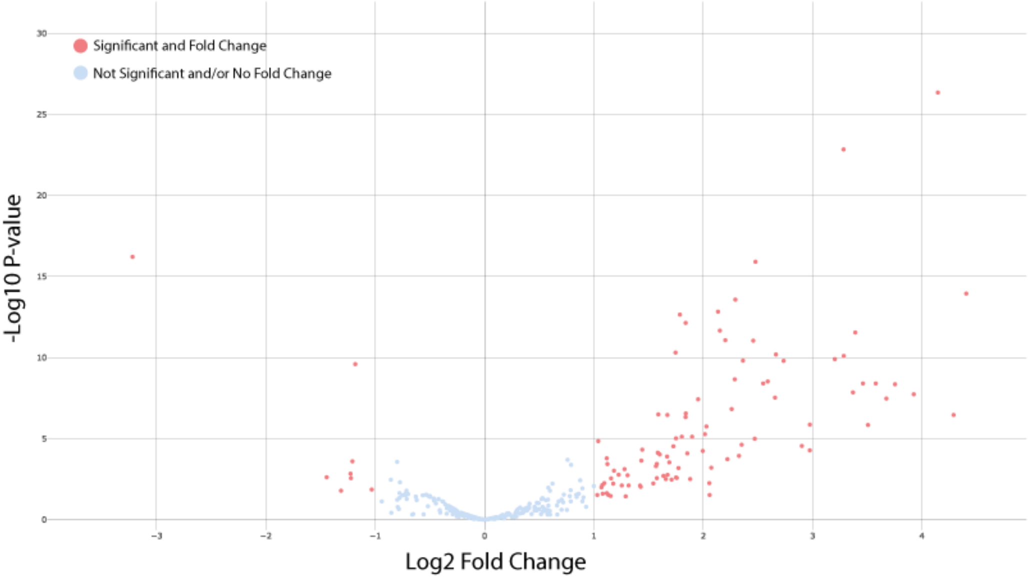

Supplementary Figure 1All differentially expressed miRNAs between TCRαβ CD8 T-LGL cells (n = 6) and healthy control TEMRA cells (n = 5).

```
5'     ...AGUGACAAUUUACAGGAAUGUAG...     3' UTR SOCS3
             ||||        ||||||
3'    UGAGUGGCUGUCGCAA---CUUACAA         miR-181a
```

Pos 1241-1247 3’ UTR SOCS3

```
5'      ...UGGAAUUUAUUAUAAGAAUGUAA...    3' UTR DUSP6
                          ||||||
3'        UGAGUGGCUGUCGCAACUUACAA        miR-181a
```

Pos 1147-1153 3’ UTR DUSP6

Supplementary Figure 2 Base pairing comparison between mature miR-181a, SOCS3 3′ UTR and DUSP6 3′ UTR putative target site is shown according to targetscan.org (www.targetscan.org).

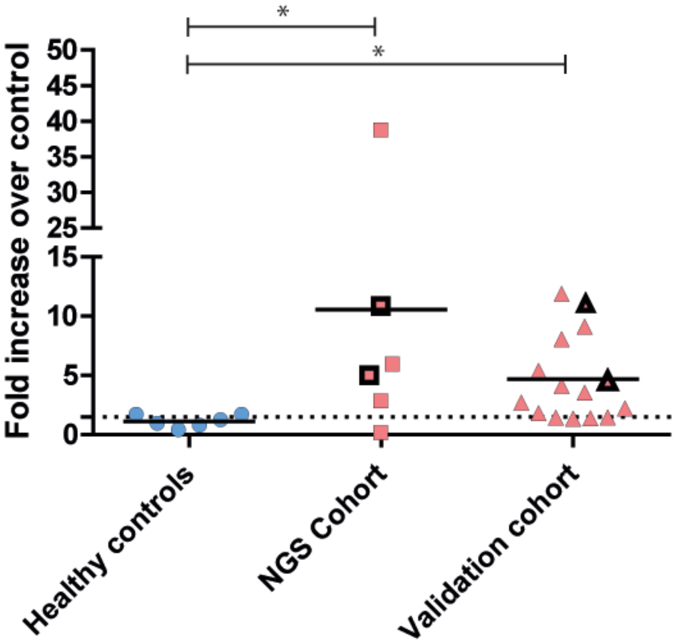

Supplementary figure 3 mRNA expression of miR-181a was determined in pure T-LGL fractions and compared to the expression in CD8 TEMRA cells. T-LGL samples with mutations in the STAT3 gene are depicted in bold.

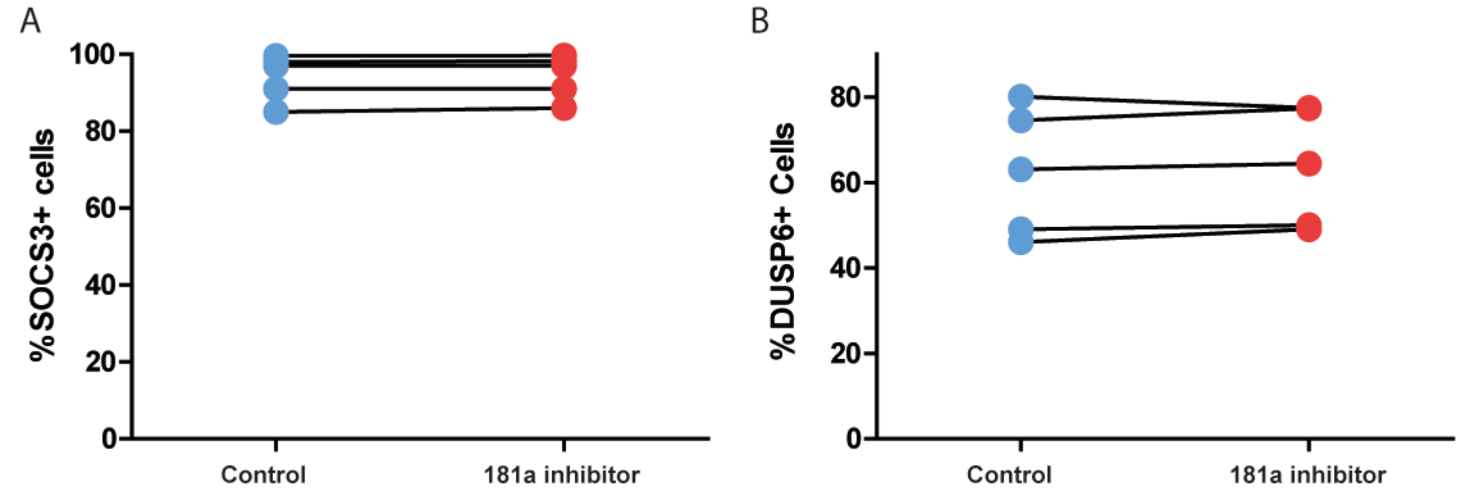

Supplementary figure 4 %SOCS3+ CD8+ T-cells in T-LGL patients 24 hours post treatment (n=5; A). %DUSP6+ CD8+ T-cells in T-LGL patients 24 hours post treatment (n=5; B). Cells were treated with a miR-181a inhibitor or non-specific miR-inhibitor.

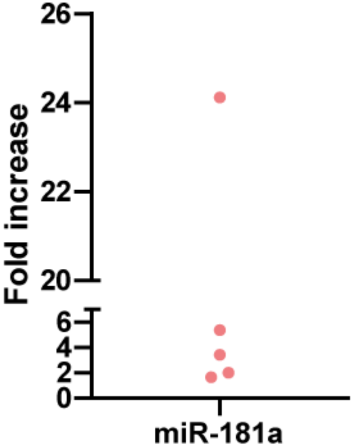

Supplementary Figure 5 mRNA expression of miR-181a 48 hours post transfection in TCRαβ CD8 T-cells (n=5). Expression is relative to the GFP- fraction.

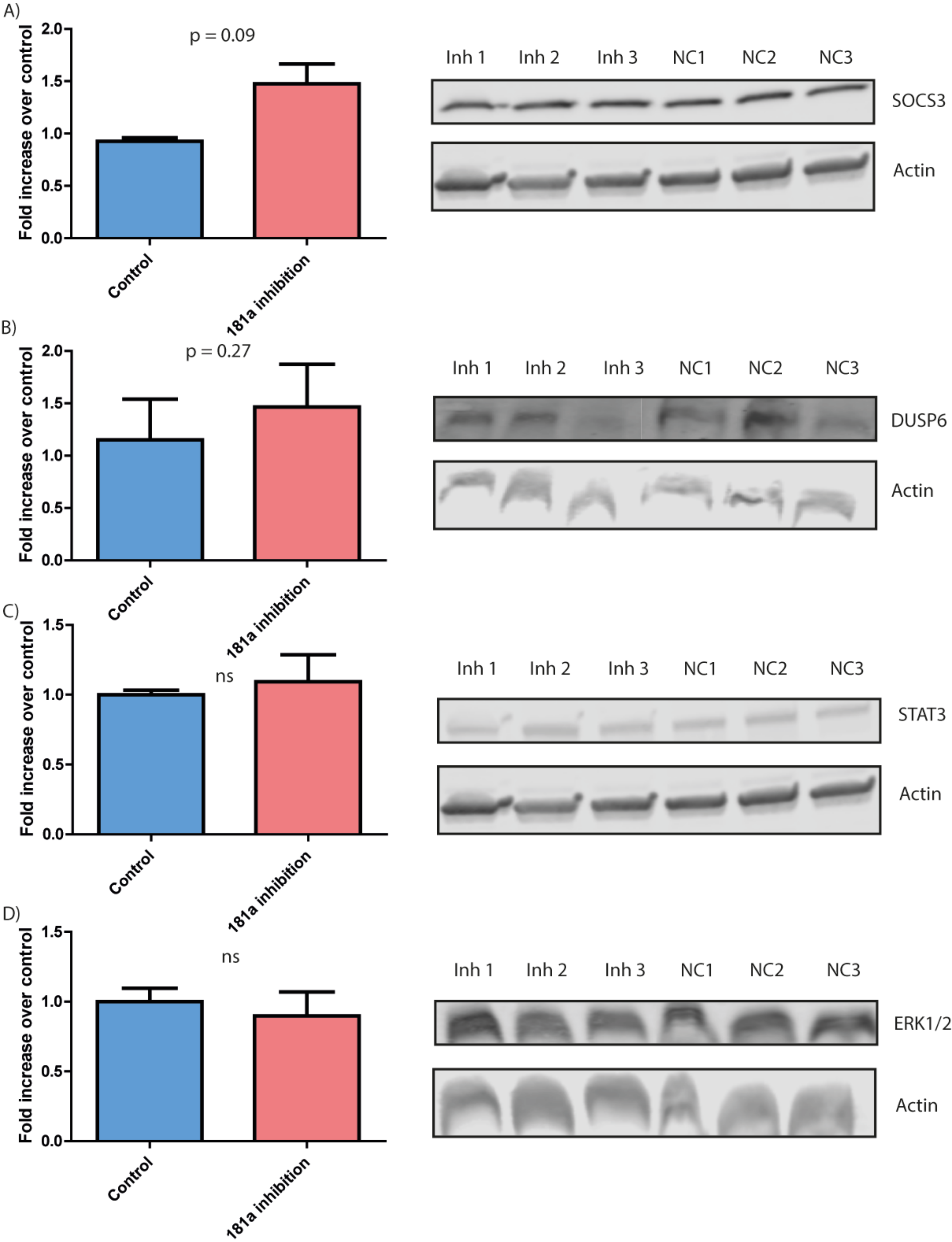

Supplementary figure 6 Westernblot results of SOCS3 (A), DUSP6 (B), STAT3 (C) and ERK1/2 (D) 48 hours post miR-181a inhibition or inhibition with a nonspecific miR-inhibitor. Graphs indicate mean plus SEM. Statistical significance was tested with the Students t-test.

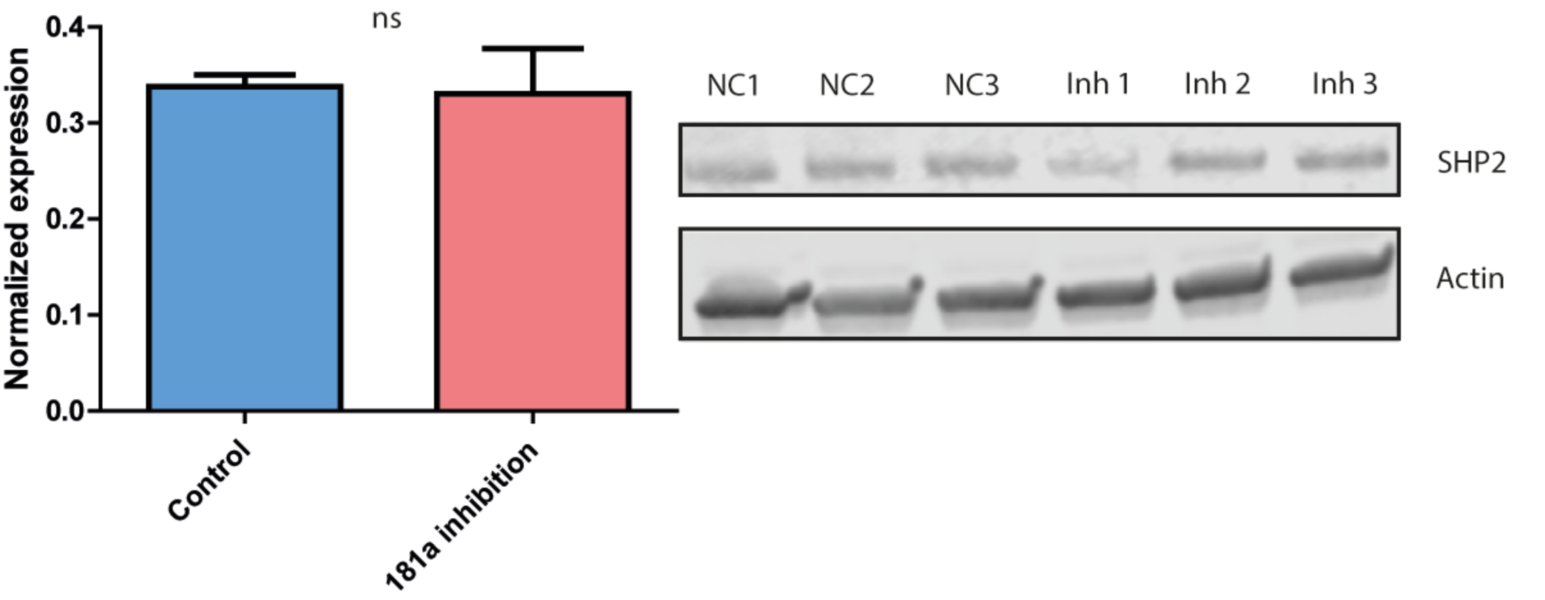

Supplementary figure 7 Westernblot of SHP2 protein 48 hours post miR-181a inhibition or inhibition with a nonspecific miR-inhibitor. Graphs indicate mean plus SEM. Statistical significance was tested with the Students t-test.

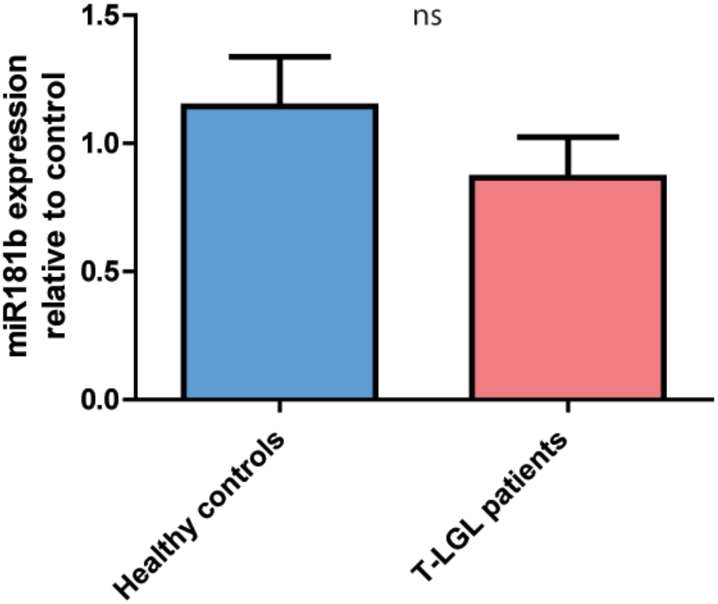

Supplementary figure 8 miR-181b expression in primary T-LGL samples (n=15) compared to healthy control CD8 TEMRA cells (n=6).Graphs indicate mean plus SEM. Statistical significance was tested with the Students t-test.

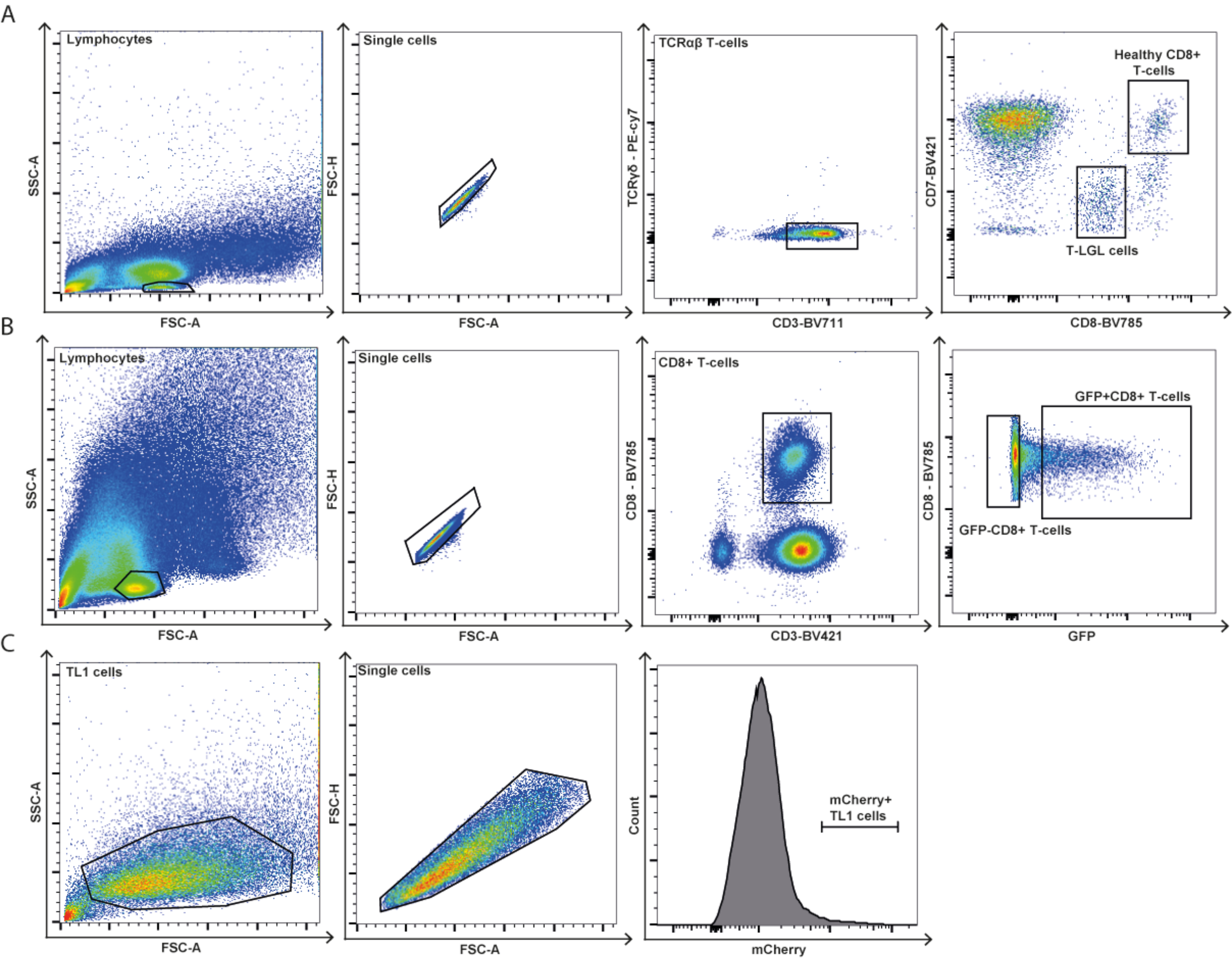

Supplementary figure 9 Gating strategy of experiments in primary T-LGL cells (A), healthy control cells (B) and the TL1 cell line (C).
