# Supplementary material for: miR-181a is a novel player in the STAT3-mediated survival network of TCRαβ+ CD8+ T large granular lymphocyte leukemia
Source: Leukemia. 2021 Dec 6;36(4):983–93. doi: 10.1038/s41375-021-01480-2 (PMC8979821; doi:10.1038/s41375-021-01480-2)
Supplement: Supplementary file 2 — Supplementary tables [file 41375_2021_1480_MOESM2_ESM.pdf]

Supplementary Table 1. Patient Characteristics

Patient	Age	Gender	Immunophenotype	Associated Disease	Symptoms	Therapy	STAT3 mutation
LGL018	72	F	CD3+,CD8+,CD2+,CD5+,CD7+,CD57+,HLADR+	None	B-symptoms	None	No
LGL028	76	F	CD3+,CD8+,CD2+,CD5het,CD7-	Unknown	Unknown	Unknown	Y640F
LGL030	38	F	CD3+,CD8+,CD2+,CD5-,CD7+,CD16-,CD57+	None	Hepato/Splenomegaly, Neutropenia	MTX	No
LGL052	61	F	CD3+,CD8dim,CD2+,CD5dim,CD7dim,CD16-,CD56+,CD57+	None	Recurrent infections	None	No
LGL141	35	F	CD3+,CD8+,CD2dim,CD7dim	Autoimmune hemolysis	Anemia	None	Y640F
LGL148	77	F	CD3+,CD8+,CD2+,CD5dim,CD7dim	Sarcoidosis, Uveitis anterior	Pancytopenia	MTX	No
LGL-MSZ-1	36	F	CD3+, CD8+, CD4-, CD45+, CD7w, CD26-, CD28het, CD197-, CD25-, CD30-, cyGranzB+, cyPerfw, CD2+, CD45RO-, CD45RA++, HLADRhet, CD16het, CD11c-, CD27-, CD5w, cyTCL1-, CD57++, CD279-, CD94-	None	Neutropenia	G-CSF	Y640F
LGL-MSZ-2	48	M	CD3+, CD8+, CD4-, CD45+, CD7+, CD26-, CD28-, CD197-, CD25-, CD30-, cyGranzB+, cyPerf-, CD2+, CD45ROhet, CD45RAhet, HLADR-, CD16het, CD11c-, CD27-, CD5+, cyTCL1-, CD57++, CD279-, CD94-	None	Neutropenia	None	No
LGL-MSZ-4	79	F	CD3+, CD8+, CD4-, CD45+, CD7-, CD26-, CD28-, CD197-, CD25-, CD30-, cyGranzB++, cyPerf+, CD2+, CD45RO-, CD45RA++, HLADR+, CD16het, CD11c-, CD27-, CD5w, cyTCL1-, CD57+, CD279-, CD94-	RA	Neutropenia	MTX	N647I
LGL-MSZ-5	49	M	CD3+, CD8+, CD4-, CD45+, CD7-, CD26-, CD28het, CD197-, CD25-, CD30-, cyGranzBhet, cyPerf-, CD2+, CD45RO+, CD45RA+, HLADR-, CD16het, CD11c-, CD27-, CD5+, cyTCL1-, CD57het, CD279-, CD94-	None	B-symptoms, Thrombopenia	Prednisone	No
LGL-MSZ-6	58	M	CD3+, CD8+, CD4-, CD45+, CD7het, CD26-, CD28-, CD197-, CD25-, CD30-, cyGranzB+, cyPerfw, CD2+, CD45RO-, CD45RA+, HLADR-, CD16-/het, CD11C-, CD27-, CD5+, cyTCL1-, CD57+, CD279-, CD94-	None	Neutropenia	None	No
LGL-MSZ-9	77	M	CD3+, CD8+, CD4-, CD45+, CD7-, CD26-, CD28-, CD197-, CD25-, CD30-, cyGranzB++, cyPerf+, CD2+, CD45RO-, CD45RA++, HLADRhet, CD16+, CD11c-, CD27-, CD5w, cyTCL1-, CD57het, CD279-, CD94-	Plasmacel dyscrasia	Anemia, Neutropenia	None	No
LGL-MZS-10	51	M	CD3+, CD8dim, CD4+, CD45+, CD7-, CD26-, CD28-, CD197-, CD25-, CD30-, cyGranzB+, cyPerfw, CD2+, CD45ROw, CD45RAw, HLADR-, CD16-, CD11c-, CD27-, CD5+, cyTCL1-, CD57++, CD279w, CD94-	None	Leukocytosis	None	No
LGL-MSZ-11	64	F	CD3+, CD8++, CD4-, CD45+, CD7+, CD26-, CD28-, CD197-, CD25-, CD30-, cyGranzB+, cyPerfhet, CD2+, CD45RO-, CD45RA+, HLADR-, C D16-, CD11c-, CD27-, CD5+, cyTCL1-, CD57++, CD279-, CD94-	Diabetus mellitus	None	None	No

LGL-UMG-1	62	M	CD3+, CD8+, CD4-, CD45+, CD7-, CD26-, CD28-, CD197-, CD25-, CD30-, cyGranzB+, cyPerfw, CD2+, CD45ROhet, CD45RA+, HLADR-, C D16-, CD11c-, CD27-, CD5w, cyTCL1-, CD57het, CD279-, CD94-	None	Neutropenia	None	No
LGL-UMG-2	69	M	CD3+, CD8+, CD4-, CD45+, CD7-, CD26-, CD28-, CD197-, CD25-, CD30-, cyGranzB+, cyPerfw, CD2+, CD45RO+, CD45RA-, HLADR-, C D16-, CD11c-, CD27-, CD5w, cyTCL1-, CD57++, CD279-, CD94-	T-LGL post Allo Tx	None	Prednisone,<br>Tacrolimus	No
LGL-UMG-3	64	F	CD3+, CD8+, CD2+,CD5+,CD7het,CD57+	T-LGL post ALLOTx due to AML	Lymphocytosis	Cyclophosphamide<br>Tacrolimus	No
LGL-UMG-7	61	V	CD3+, CD8+, CD4-, CD45+, CD7w, CD26-, CD28-, CD197-, CD25-, CD30-, cyGranzB+, cyPerf het, CD2+, CD45ROhet, CD45RA+/het, HLADR-, C D16w, CD11c-, CD27het, CD5+, cyTCL1-, CD57+, CD279-, CD94-	Sarcoidosis<br>Arthritis<br>Eczema	Lymphopenia<br>Opportunistic infections	Mometason	No
LGL-UMG-8	73	F	CD3+, CD8+, CD4-, CD45+, CD7-, CD26-, CD28-, CD197-, CD25-, CD30-, cyGranzB+, cyPerf-, CD2w, CD45RO-, CD45RA+, HLADR+, CD16het, CD11c+, CD27-, CD5w, cyTCL1-, CD57+, CD279-, CD94-	RA<br>Bronchial asthma	Neutropenia	MTX<br>Prednison<br>Cyclophosphamide<br>Cyclosporine A	No
LGL-UMG-9	61	F	CD3+, CD8+, CD4-, CD45+, CD7het, CD26-, CD28het, CD197-, CD25-, CD30-, cyGranzB+, cyPerf het, CD2+, CD45RO het, CD45RA+, HLADR het, C D16het, CD11c-, CD27-, CD5het, cyTCL1-, CD57+, CD279-, CD94-	RA	Neutropenia	Cyclosporine A<br>MTX	No
LGL-UMG-10	56	F	CD3+, CD8+, CD4-, CD45+, CD7-, CD26-, CD28-, CD197-, CD25-, CD30-, cyGranzB++, cyPerf-/w, CD2+, CD45RO-, CD45RA+, HLADRhet, C D16het, CD11c+, CD27-, CD5-, cyTCL1-, CD57het, CD279-, CD94+	Rosacea<br>Myositis	Neutropenia	Cyclosporine A<br>Prednison<br>MTX	No

AML = acute myeloid leukemia, AIHA = Autoimmune hemolytic anemia, CSA = Cyclosporine A,G-CSF = granulocyte colony stimulating factor , Het = heterogeneous, MTX = methotrexate, RA = Rheumatoid Arthritis, Tx = transplantation, W=weak

Supplementary table 2. Antibodies

Antibody	Clone	Company	Titer
**CD2 - FITC**	TS1/8	Biolegend	5 µl
**CD3 - PerCP-Cy5.5**	SK7	BD Biosciences	10 µl
**CD3 - BV421**	UCHT1	Biolegend	0.5 µl
**CD5 - PE-Cy7**	L17F12	BD Biosciences	5 µl
**CD7 - BV421**	M-T701	BD Biosciences	5 µl
**CD8 - APC-H7**	SK1	BD Biosciences	5 µl
**CD8 - BV785**	RPA-T8	Biolegend	0.5 µl
**CD57 - BV605**	QA17A04	Biolegend	2.5 µl
**CD45RA - APC-H7**	HI100	BD Biosciences	5 µl
**TCRαβ - APC**	IP26	eBiosciences	5 µl
**CCR7 - PE-CF594**	150503	BD Biosciences	1 µl
**TCRγδ - PE-Cy7**	11F2	BD Biosciences	1 µl
**pSTAT3 - PE**	4-P-STAT3	BD biosciences	20 ul
**pERK1/2 - PE**	20A	BD biosciences	5 ul
**DUSP6 - AF647**	F12	Santa Cruz Biosciences	1 ul
**SOCS3**	Polyclonal	Abcam	1 ul
**Annexin V - APC**	n/a	BD biosciences	0,5 ul
**7-AAD**	n/a	BD biosciences	0,25 ul
**CD95**	EOS9.1	eBioscience	n/a
**Goat anti Rabbit IgG - AF 647**	Polyclonal	ThermoFisher	1/100

Supplementary table 3. SOCS3 and DUSP6 WT and MT 3’ UTRs

Sequence name	Sequence 5' -> 3'
SOCS3_181_WT_FW	CTAGC CACTGATCAGTGACAATTTACAGGAATGTAGCAGCGATGGAATTACCTGGAACA G
SOCS3_181_WT_RV	TCGAC TGTTCCAGGTAATTCCATCGCTGCTACATTCCTGTAAATTGTCACTGATCAGTG G
SOCS3_181_MT_FW	CTAGC CACTGATCAGTGACAATTTACAGGACCATAGCAGCGATGGAATTACCTGGAACA G
SOCS3_181_MT_RV	TCGAC TGTTCCAGGTAATTCCATCGCTGCTATGGTCCTGTAAATTGTCACTGATCAGTG G
DUSP6_181_WT_FW	CTAGC TTTAGAGCTGGAATTTATTATAAGAATGTAAAACCTTAAATTATTAATAAATAA G
DUSP6_181_WT_RV	TCGAC TTATTTATTAATAATTTAAGGTTTTACATTCTTATAATAAATTCCAGCTCTAAA G
DUSP6_181_MT_FW	CTAGC TTTAGAGCTGGAATTTATTATAAGACCATAAAACCTTAAATTATTAATAAATAA G
DUSP6_181_MT_RV	TCGAC TTATTTATTAATAATTTAAGGTTTTATGGTCTTATAATAAATTCCAGCTCTAAA G

ID	log2FC	padj	CD8TEMRA1	CD8TEMRA2	CD8TEMRA3	CD8TEMRA4	CD8TEMRA5	LGL0L18	LGL028	LGL030	LGL052	LGL141	LGL148
hsa-let-7a-5p	2,293215414	2,66E-14	15,6441732	15,23143744	15,5528454	15,0450386	15,12749655	16,55795	16,48646	16,59005	16,68979	16,36306	16,50987
hsa-let-7f-5p	2,028662373	1,78E-06	15,1162183	14,56169254	15,2761645	14,38161874	14,78414038	15,97969	16,02826	15,9068	16,29747	15,60363	15,72753
hsa-let-7b-5p	1,152397357	0,034329	14,7029776	14,79639005	14,6190981	14,54772899	14,1671919	15,16395	15,19313	15,78639	14,31426	14,57427	15,51394
hsa-miR-26a-5p	2,220498689	0,000185	13,8728125	13,52524309	14,3562864	13,41529429	13,89607192	14,86614	15,72386	14,44904	15,57065	14,29767	14,51494
hsa-miR-26b-5p	1,71013837	0,003337	13,8859139	13,6576905	14,4520973	13,47241227	13,94703752	14,83851	15,49287	14,51486	15,20075	13,89506	14,4581
hsa-miR-92a-3p	1,839808595	2,79E-07	13,4374304	14,01785671	14,2622125	13,82858905	13,80822609	14,75276	15,04059	14,53173	15,11375	14,71531	14,97759
hsa-miR-21-5p	2,259085646	1,50E-07	13,7794935	13,47861671	14,0185138	13,30757719	13,53601241	14,54062	15,53942	14,81552	14,66012	14,57885	14,48302
hsa-miR-181a-5p	4,28915194	3,48E-07	12,8585779	11,96473698	12,7765467	11,93122015	12,43979308	14,38574	14,73829	14,21582	12,91051	14,9227	15,34124
hsa-let-7g-5p	1,635047292	0,001978	13,6786832	13,5184734	14,1590086	13,30989135	13,65284014	14,58167	15,10436	14,43914	14,90389	13,67647	14,10788
hsa-let-7i-5p	2,477305818	1,23E-16	13,3702411	13,38715148	13,5590584	13,14191978	13,11713435	14,51417	14,89121	14,52372	14,8839	14,47234	14,18625
hsa-miR-155-5p	2,072887786	0,000619	13,7243373	12,80925135	13,6320246	12,63247489	13,18231931	14,76231	14,06311	14,6389	13,66208	14,78879	13,93242
hsa-miR-142-5p	3,753129031	4,42E-09	11,757176	11,61448956	12,5084811	11,51882443	12,08279433	13,87552	14,5689	13,07506	14,21709	13,28703	13,43505
hsa-miR-146a-5p	3,202466261	1,25E-10	12,1519601	11,76056598	12,2332175	11,65202359	11,86914131	13,88641	13,61144	12,68893	13,9212	12,78847	13,986
hsa-miR-25-3p	1,803407165	7,65E-06	12,2990166	12,11631691	12,5110115	11,92070352	12,03644173	13,00875	13,62301	13,31051	13,27704	12,6563	12,61252
hsa-miR-221-3p	2,350489577	2,39E-05	10,8864972	11,45021342	11,1668412	11,27867782	10,86363325	12,10857	13,12482	12,56811	12,62781	11,50562	11,4757
hsa-miR-181b-5p	2,973714897	5,25E-05	10,9294033	10,09308204	11,0062992	10,05086921	10,58634193	11,26195	12,24692	12,32859	10,69094	12,3181	12,92831
hsa-let-7d-5p	2,200969546	8,41E-12	11,1106252	10,94962922	11,1345834	10,67723776	10,76735002	12,02296	12,35734	12,20382	12,31296	11,84964	11,58222
hsa-miR-28-3p	2,456906218	9,07E-12	10,3387896	10,83584331	11,1590162	10,70291352	10,70546483	11,76535	12,23806	12,06026	12,12291	12,23336	11,80917
hsa-miR-30d-5p	2,547483892	3,92E-09	10,4434608	10,55748901	10,779041	10,41905534	10,4013285	11,48959	12,36314	11,41097	12,44302	11,45455	11,31863
hsa-miR-22-3p	3,282498408	1,45E-23	10,1692292	10,14509732	10,431236	10,05485629	10,15792518	11,64767	12,27502	11,51628	12,11252	11,91314	11,3313
hsa-miR-191-5p	1,441668579	4,79E-05	10,5376752	10,84741288	11,206049	10,59708679	10,68110326	11,53173	11,55863	11,23139	11,76949	11,90185	11,24519
hsa-miR-196a-5p	4,404583746	1,13E-14	9,56023183	9,115738425	9,21347444	9,15074601	9,03655594	11,64858	11,9291	10,6418	9,987713	11,17934	11,85095
hsa-miR-320b	-1,226207063	0,001451	11,0480928	11,65855309	11,4108277	10,98233299	10,5164082	10,35114	10,61091	10,54275	10,72625	10,30387	10,58754
hsa-miR-98-5p	1,749866974	9,58E-06	10,3602128	9,90000106	10,6071381	9,745092941	10,08998123	11,12509	11,10434	11,15101	11,46621	10,83388	10,873
hsa-miR-30e-5p	3,459721869	3,92E-09	9,62498066	8,905774747	9,50475242	8,854991267	9,106720869	11,16086	11,80433	10,27493	11,14729	10,39047	10,48864
hsa-miR-140-3p	2,014861422	5,28E-06	9,69174638	10,0354742	10,3602778	9,881946239	9,920631775	11,01848	11,74493	10,79951	11,16488	10,69233	10,46186
hsa-miR-16-5p	3,368628151	1,40E-08	9,10742697	8,970230538	9,64418043	8,896525369	9,317040975	11,31013	11,62756	10,67022	10,87816	10,68569	9,52782
hsa-miR-192-5p	2,900809269	2,81E-05	8,67947057	9,139197307	9,9822734	8,989593469	9,520870186	11,07399	11,45221	9,950666	11,23582	10,41615	10,51087

ID	log2FC	padj	CD8TEMRA1	CD8TEMRA2	CD8TEMRA3	CD8TEMRA4	CD8TEMRA5	LGL0L18	LGL028	LGL030	LGL052	LGL141	LGL148
hsa-miR-101-3p	3,924529404	1,83E-08	8,37762734	8,478947419	9,2936626	8,538119879	8,946626989	10,48613	11,73561	10,03087	11,14519	9,675404	10,33156
hsa-miR-186-5p	3,674038967	3,37E-08	8,37991854	8,491207471	9,3308471	8,469306038	9,041392497	10,62764	11,47178	10,08797	11,02761	10,08181	9,945896
hsa-miR-29a-3p	3,577016721	3,92E-09	8,48792764	8,207457126	8,30058762	8,019398271	8,17947817	9,697062	11,12262	9,358776	10,40275	8,937754	9,426895
hsa-miR-148a-3p	3,389890374	2,81E-12	7,83110599	7,975954503	8,58067558	7,998232611	8,311537792	9,644979	10,42905	9,539066	10,2879	9,429518	9,543245
hsa-miR-1307-3p	1,745959861	4,92E-11	8,88678726	9,019249911	9,16810157	8,902067501	8,783386474	9,486325	9,880697	9,967219	10,22229	9,714724	9,827202
hsa-miR-30a-3p	1,897748641	7,66E-06	8,73026129	9,187920558	8,67131586	8,927809458	8,375442049	9,727936	10,30529	9,891305	9,900059	9,477022	9,263433
hsa-miR-222-3p	2,326100796	0,000115	7,86205803	8,71779695	8,81924586	8,533355068	8,403498625	9,663439	10,31362	9,787225	10,14368	8,730939	9,008337
hsa-miR-181a-2-3p	2,057289925	0,029833	8,92301681	7,825149115	8,39694097	7,824083886	8,156437095	7,702339	8,444906	9,652564	8,060605	9,59106	10,5763
hsa-miR-10a-5p	3,505893502	1,43E-06	8,12597485	7,335763813	7,53606623	7,335042034	7,437782603	8,363055	9,528098	8,088705	8,916043	10,59395	9,148958
hsa-miR-7-5p	1,279675701	0,000762	8,55582224	8,847638549	9,12870752	8,505308732	8,731549001	9,52218	9,896922	9,601368	9,13853	9,292822	9,034344
hsa-miR-30e-3p	1,225911349	0,00168	8,85696689	8,765603819	8,97384772	8,630821528	8,582535281	9,480724	9,939026	9,212304	9,619101	8,991316	8,918596
hsa-miR-10b-5p	4,144940824	4,49E-27	7,29462885	7,273846636	7,27309966	7,281059926	7,292318613	8,826168	9,50529	9,083876	9,155716	8,569341	9,950992
hsa-miR-197-3p	-3,219703398	6,08E-17	8,8870133	9,855112998	9,26428843	9,475331488	8,752388769	7,500078	7,853236	7,5762	7,815408	7,530637	7,350418
hsa-miR-28-5p	1,290099582	0,036382	7,55194233	8,349005889	8,82807392	8,276776408	8,4940453	8,747515	9,745684	9,070304	9,138783	8,368247	8,834028
hsa-miR-361-3p	2,589086674	2,97E-09	7,39625945	8,017988157	7,85385779	7,917606609	7,598725916	8,677165	9,727365	8,890431	9,283452	9,084295	8,567751
hsa-miR-142-3p	3,284548123	7,85E-11	7,18542889	7,499853348	7,17098696	7,461558868	7,377512883	9,149514	9,866492	8,177559	9,044036	8,290185	8,611528
hsa-miR-425-5p	1,673284181	0,001712	7,41676105	8,495274891	8,05251895	8,304799838	7,741346282	8,794208	9,134979	8,727387	9,298273	9,230574	8,253646
hsa-miR-23a-3p	1,542627302	0,005762	8,06444627	8,127063977	8,01438304	7,875934227	7,613257884	8,737901	9,216162	8,472804	9,237216	7,501351	8,677292
hsa-miR-107	1,094368749	0,005541	7,75161399	8,560601886	8,34585504	8,147434263	7,925301974	8,527966	9,120558	8,77644	8,920259	8,682164	8,435371
hsa-miR-361-5p	1,726291655	2,97E-05	8,0987132	7,867501745	7,67151733	8,004808291	7,677233379	8,558682	9,276702	8,733648	9,029058	7,974288	8,703499
hsa-miR-24-3p	1,657465431	0,003128	7,99401114	8,137453933	7,75303498	7,713220576	7,509525619	8,754312	9,21895	8,481607	9,19621	7,531665	8,361099
hsa-miR-629-5p	2,656240504	2,95E-08	7,01554725	7,218857583	7,46771546	7,153497143	7,300617184	8,330849	9,322861	8,541839	8,145189	8,894054	7,668611
hsa-miR-941	2,150979337	2,16E-12	7,48923145	7,667580137	7,43494019	7,693181883	7,291616152	8,294149	8,666257	8,675764	8,848378	8,934026	8,203286
hsa-miR-148b-3p	1,575608773	0,000362	7,1505354	7,810810629	8,01616355	7,68524926	7,67353791	8,201044	9,087759	8,2663	8,747635	8,292778	8,298812
hsa-miR-27b-3p	1,994885611	5,73E-05	7,08011257	7,281942611	7,95601647	7,256319593	7,604414605	8,522876	9,07851	8,227537	8,825339	7,905324	8,187535
hsa-miR-23b-3p	1,575329352	0,002738	6,96104726	7,762172317	7,53670786	7,524817538	7,24423129	8,13155	8,695509	7,98705	8,469275	7,251659	8,554743
hsa-miR-181c-5p	2,734577759	1,59E-10	6,7987358	7,028540269	6,77162129	6,96669082	6,79583423	7,868355	8,12946	7,719586	9,094837	8,235334	7,952987
hsa-miR-369-3p	2,974557241	1,37E-06	6,65846911	6,619642043	6,61863813	6,629322343	6,644381339	7,7626	9,205693	7,970367	8,023336	6,601488	7,934221

ID	log2FC	padj	CD8TEMRA1	CD8TEMRA2	CD8TEMRA3	CD8TEMRA4	CD8TEMRA5	LGL0L18	LGL028	LGL030	LGL052	LGL141	LGL148
hsa-miR-769-5p	2,362537639	1,53E-10	6,84908277	6,823476967	7,40212292	6,832380018	7,101081045	8,156754	8,718469	7,958178	8,364502	8,152338	7,876525
hsa-miR-152-3p	1,427450283	0,009448	6,89513848	7,130186726	7,93054145	7,031400776	7,552259305	7,668493	8,182284	8,378527	8,608163	8,130834	7,498333
hsa-miR-9-5p	2,054971069	0,005541	7,65920618	6,658194715	6,65720607	6,667728424	6,682561817	7,609515	8,915749	7,272542	7,995287	8,432694	6,806547
hsa-miR-4446-3p	-1,222074804	0,002738	7,44598712	8,360876459	8,16827085	8,296207558	7,75716858	7,432776	7,068321	7,338216	7,244013	7,81706	7,354534
hsa-miR-3615	1,129879819	0,029171	7,78518098	7,514406462	6,81064398	7,503026447	6,834348787	7,814342	8,352023	8,139268	8,068771	7,531927	7,662794
hsa-miR-128-3p	1,689126544	0,000292	6,75761047	7,175460602	7,28375387	7,062922005	7,095028236	7,840352	8,622143	7,846957	8,249356	7,343961	7,361455
hsa-miR-484	1,433618553	0,000227	6,80951526	7,068087446	7,65050054	7,101066473	7,254995046	7,634838	8,262466	7,585932	7,993781	8,146468	8,030944
hsa-miR-15a-5p	2,664346889	6,43E-11	6,62553836	6,597493454	6,59648144	6,607251691	6,622431199	8,256293	8,532521	7,568532	7,992635	7,767411	7,099081
hsa-miR-27a-3p	1,88022445	0,003123	6,64046631	7,224776224	6,61156509	7,153406907	6,6373757	7,65524	8,444963	7,487832	8,51225	6,594369	7,578327
hsa-miR-30a-5p	1,757667423	0,002662	6,71519005	6,685433116	7,40476353	6,623033568	6,701752007	7,713437	8,575101	7,445062	7,831402	6,750524	7,712546
hsa-miR-29c-3p	2,470322034	1,01E-05	6,48615764	6,468110654	6,45548736	6,466860803	6,482880065	7,463001	8,607086	7,168304	8,204542	6,77249	7,10269
hsa-miR-19b-3p	1,750599488	0,002486	6,54357999	6,502953827	7,09416432	6,513079619	6,866362361	7,424561	8,494176	7,223287	7,944206	6,926257	7,008608
hsa-miR-127-3p	2,287707667	2,19E-09	6,49190823	6,462377407	6,46131104	6,472657055	6,488638335	7,01973	8,12383	7,462102	7,594422	7,154712	8,053268
hsa-miR-141-3p	1,583776724	7,43E-05	6,53622396	6,746971637	6,73691793	6,655420165	6,962706295	7,861226	8,047447	6,926108	7,578092	7,382093	7,180752
hsa-miR-185-5p	1,07978064	0,024723	6,56026982	7,118445664	7,15313875	6,762817656	6,93838054	7,277524	8,235326	7,474579	7,155921	7,256232	7,13397
hsa-miR-130b-3p	1,83766818	4,67E-07	6,50538355	6,804089777	6,47494703	6,721240254	6,502130552	7,069757	8,150454	7,604915	7,230427	7,5146	7,461745
hsa-miR-140-5p	1,307651614	0,001818	6,54791026	6,775302361	7,14319261	6,714667654	6,896350407	7,334361	8,169389	7,187495	7,571539	7,349813	7,146538
hsa-miR-432-5p	1,11758995	0,023202	6,52896395	6,816875807	7,10374041	6,922618062	6,871596657	7,18674	7,785393	7,49219	7,472644	6,480886	7,817706
hsa-miR-532-5p	2,13496612	1,47E-13	6,46226175	6,432446189	6,43136955	6,442824937	6,458960327	7,373168	7,761488	7,380062	7,920046	7,050314	7,504259
hsa-miR-199a-3p	1,772787426	0,00065	6,39910711	6,592795045	6,36743939	6,623552723	6,395724148	7,089774	8,150728	7,143847	7,646757	6,348562	7,250097
hsa-miR-374a-3p	1,317147376	0,007766	6,42697843	6,664760544	6,93646198	6,520014723	6,589487394	7,581658	7,96907	6,908421	7,432628	6,651228	6,866923
hsa-miR-16-2-3p	1,952299975	3,75E-08	6,38127505	6,350540343	6,34943002	6,361241974	6,37787311	7,612022	7,752575	7,234285	7,01737	7,553834	6,668672
hsa-miR-660-5p	1,853848224	8,09E-05	6,31465631	6,283150659	6,28201202	6,294123537	6,311170242	6,856012	7,820491	6,966045	7,585064	6,262541	7,377173
hsa-miR-21-3p	1,83881945	7,24E-13	6,36970344	6,35114768	6,33810299	6,349817429	6,366324918	6,985417	7,568999	7,119	7,089021	7,57295	7,280427
hsa-miR-196b-5p	1,421718254	0,007812	6,90683813	6,303747982	6,30261765	6,314641165	6,331565401	6,79102	7,957886	7,446838	6,980642	6,529762	6,88989
hsa-miR-324-5p	1,785779362	2,22E-13	6,34780886	6,316688788	6,31556433	6,327525893	6,344364822	6,907135	7,323276	6,931527	7,402269	7,365998	7,423857
hsa-miR-454-3p	1,671219795	3,48E-07	6,29044567	6,258669865	6,25752135	6,269737546	6,286930012	6,999476	7,727705	6,913545	7,148652	6,748499	6,969675
hsa-miR-143-3p	1,669134441	0,000128	6,26500437	6,232945311	6,23178642	6,244112452	6,261457719	6,669645	7,656198	7,088498	7,218476	6,211966	7,280511

ID	log2FC	padj	CD8TEMRA1	CD8TEMRA2	CD8TEMRA3	CD8TEMRA4	CD8TEMRA5	LGL0L18	LGL028	LGL030	LGL052	LGL141	LGL148
hsa-miR-412-5p	1,601694787	9,43E-05	6,25171145	6,219503532	6,23100076	6,230722965	6,248148523	6,576356	7,598801	7,059443	6,793845	6,462074	7,421005
hsa-miR-29b-1-5p	1,58849615	3,21E-07	6,26751123	6,235488226	6,23433068	6,246642541	6,263968461	6,933174	7,469659	7,153815	7,260065	6,585373	6,759882
hsa-miR-382-5p	1,567477098	0,000499	6,22795334	6,195480848	6,19430681	6,206793201	6,224361475	6,473966	7,472724	7,446946	6,962722	6,174225	7,031439
hsa-miR-877-5p	-1,209081191	0,00025	6,40220233	7,145329783	7,30835922	7,006407913	7,092003933	6,510182	6,207703	6,333857	6,269475	6,63583	6,317645
hsa-miR-199b-3p	1,176527682	0,005945	6,16807212	6,258404151	6,13373349	6,258813385	6,164407236	6,517876	7,437601	6,617203	6,920737	6,11323	6,792202
hsa-let-7f-1-3p	-1,444188156	0,002408	7,10525303	7,311314798	6,07671927	7,097105791	6,107973976	6,097096	5,987093	6,033671	6,018357	6,332613	6,038785
hsa-miR-409-3p	1,254806535	0,007736	6,12572947	6,092117899	6,09090213	6,103830379	6,122013026	6,177738	7,451224	6,843574	6,814142	6,08326	6,596029
hsa-miR-30b-5p	1,076610034	0,007235	6,14325904	6,246034408	6,10863867	6,222843587	6,139564417	6,586266	7,373186	6,434818	6,843043	6,465753	6,302656
hsa-miR-126-5p	1,183515699	0,000947	6,13058843	6,097034586	6,09582094	6,108726752	6,126878288	6,684927	7,218848	6,585132	6,622549	6,08819	6,84299
hsa-miR-323a-3p	1,156758321	0,002824	6,11665975	6,082951011	6,08173169	6,094697599	6,112932675	6,464515	7,072338	7,119982	6,610807	6,060869	6,492147
hsa-miR-194-5p	1,123043479	0,000367	6,11997288	6,086304849	6,08508707	6,098036865	6,116250144	6,451357	7,118601	6,210032	6,762669	6,747775	6,583458
hsa-miR-1307-5p	1,11693554	0,000163	6,12084997	6,087191651	6,08597422	6,098920268	6,117128301	6,567111	7,057665	6,327171	6,921408	6,445775	6,568697
hsa-miR-130a-3p	1,068722598	0,010108	6,08534218	6,051285478	6,0500534	6,063154328	6,08157706	6,105064	6,980986	6,609326	6,533369	6,02897	7,059422
hsa-miR-589-5p	1,038997836	1,42E-05	6,10846365	6,088052388	6,07348547	6,086468637	6,104730701	6,845288	6,860399	6,551661	6,450463	6,339514	6,626044
hsa-miR-29b-3p	1,030357325	0,029833	6,05941571	6,025068652	6,02382593	6,037039542	6,055618859	6,24629	7,297399	6,297858	6,766602	6,002559	6,285307
hsa-miR-1270	-1,312988462	0,016261	5,99645636	5,961414002	7,40106954	5,973629218	6,893023475	5,969658	5,87856	5,965511	5,839772	5,938439	5,920737
hsa-miR-1260b	-1,182805913	2,53E-10	6,62710141	6,865164709	6,64550401	6,60946175	6,4459092	6,014342	6,025341	6,08862	6,096645	6,031383	6,015385
hsa-miR-4525	-1,031994409	0,01377	7,0841879	6,432166184	5,92785275	6,317349977	5,960616731	5,833809	5,834347	5,908491	5,946511	5,905924	5,888037

Supplementary table 4. Differentially expressed microRNAs between T-LGL cells and healthy control TEMRA CD8 Lymphocytes

ID	baseMean	log2FoldChange	padj	TEMRA1	TEMRA2	TEMRA3	TEMRA4	TEMRA5	TEMRA6	LGL030	LGL037	LGL141	LGL148	LGL018	LGL028
NR6A1	1330,732668	2,586567865	0,000952	8,5732743	9,3552049	8,5861893	7,9861691	8,4989955	9,4669995	11,141	10,062	10,72	11,089	10,198	9,8936
BNC2	3687,081059	2,386572218	0,007331	9,2167443	10,194149	9,0720256	10,990655	9,5615082	11,297438	12,902	11,744	12,469	9,9632	10,676	11,809
POLA1	2018,669725	2,381038769	0,000186	9,1066112	10,573822	9,2865685	9,015148	9,0254084	9,9152257	11,352	9,9423	11,381	9,9281	11,402	11,123
CDK8	1325,062892	2,259081186	6,13E-05	8,1664509	10,123555	8,8904049	8,9265159	8,6215273	9,1999165	10,607	9,9389	10,153	10,7	10,224	10,734
KCNQ5	4073,369472	2,186438147	0,00011	10,021072	10,363431	11,441292	10,47144	10,763758	11,194164	12,46	11,213	11,448	11,917	11,988	12,2
EYA3	2878,22666	2,172623036	1,35E-05	10,223206	10,573438	9,2305612	9,9495498	10,358962	11,096695	11,568	11,446	11,73	11,697	11,645	11,717
PIK3R3	1358,704198	2,069584264	8,36E-06	9,6257477	8,9876015	8,72837	10,157632	8,9770157	8,8051901	10,9	10,685	10,734	10,648	10,645	10,088
MTX3	1034,327766	1,956769951	0,001778	7,9180497	9,2806308	8,6320139	7,9126176	8,7671174	9,8743348	10,569	9,9812	10,728	10,351	9,8138	9,8378
ZNF449	667,342093	1,880015214	0,029564	7,3186074	8,9108322	7,3837872	8,3881767	7,1301411	8,908834	7,3516	10,089	9,2026	9,5441	9,7235	9,5322
ATP11C	1544,064788	1,867680293	0,000867	9,7465767	10,02234	8,4391008	9,6499298	9,2250136	9,9414862	11,258	10,064	10,423	10,647	10,234	10,789
ACTA2	1378,510509	1,829245281	0,012245	10,042355	8,1907732	8,3956377	8,7716926	9,5883933	9,9959993	10,53	10,687	9,7005	10,762	8,6796	10,576
RAB27B	1105,859415	1,812241979	0,011997	7,9395366	10,119856	7,988459	9,1115578	9,1248215	8,6481229	9,9681	9,8524	10,708	10,563	10,796	9,8414
ZDHHC21	1337,967004	1,705419079	0,023307	8,1966848	10,013261	8,288154	9,8492049	8,1404315	10,092502	10,489	10,035	11,028	9,8427	10,393	10,523
LCLAT1	2905,289287	1,691454008	0,000434	10,823036	10,891316	10,375301	10,983914	10,445337	10,050311	11,94	10,627	11,618	11,105	11,467	11,877
HNRNPH1	19647,48047	1,676981159	0,000344	13,317713	13,424787	12,827231	13,374319	13,57686	13,743293	14,41	13,667	15,127	14,608	15,026	14,358
HOMER1	1327,167582	1,643921063	0,032443	8,1380047	8,0885416	9,2261859	10,264274	9,9128615	9,2752658	11,041	10,089	10,167	10,288	10,13	9,7451
CAMK2G	1354,850389	1,611634963	0,004262	8,6426337	8,9062539	9,1252441	9,49141	10,373326	9,833809	10,864	10,116	9,8178	10,985	9,776	10,683
ADAT2	938,4798811	1,608540279	0,041034	7,755277	9,4545046	9,7255387	7,749389	9,0823865	8,2728103	10,072	9,7441	10,594	9,8711	10,472	9,963
RFX3	7453,787104	1,604680563	0,002405	12,100472	12,018943	11,602674	11,944155	11,568344	12,606663	13,165	12,009	13,792	13,009	12,839	12,927
PDE5A	1785,201337	1,546344173	0,014135	9,7735849	8,5571106	9,9790475	10,150116	10,019509	10,568291	11,177	10,802	11,087	11,709	10,542	10,784
LPCAT1	25032,03724	1,493298517	0,012204	13,213006	13,62042	14,415239	13,924141	13,517398	13,804481	13,832	15,444	13,544	14,563	13,485	14,874
GDI1	4159,272249	1,476167425	4,88E-11	11,289065	11,510786	11,389068	10,994122	11,50118	11,263007	12,151	12,074	12,224	12,518	12,311	12,365
KAT2B	6407,234407	1,464264176	0,000316	11,982714	11,625096	12,261039	11,756376	11,843653	12,006265	12,797	11,833	12,619	12,774	12,676	13,217
SOX6	4097,508613	1,389350164	0,032939	11,351108	11,785998	11,076426	10,935096	10,743876	11,231161	12,587	10,671	12,308	12,559	12,27	11,534
ANKFY1	4339,76187	1,382980343	0,001879	11,013102	11,83961	11,439025	11,332663	10,383716	11,806404	11,977	12,602	12,494	12,039	11,757	12,215
PAPOLG	2179,460246	1,37872871	0,009331	10,177543	10,486743	9,1037419	10,968464	10,709572	10,077326	10,488	11,258	11,698	11,349	11,418	10,947
KATNBL1	2665,278752	1,325097301	0,015169	10,145953	11,176357	11,202793	10,254508	10,536133	10,327452	11,02	11,328	10,964	11,107	10,26	11,868
CEP97	1618,789304	1,291460692	0,022706	8,565175	9,6721798	10,094978	10,06099	10,497132	10,278268	11,08	11,066	11,157	10,205	10,849	10,442
AP5M1	3777,118596	1,290509225	0,000106	11,660182	11,485743	11,133934	10,59406	11,366216	11,116591	12,15	12,126	12,417	11,582	11,957	12,038

ID	baseMean	log2FoldChange	padj	TEMRA1	TEMRA2	TEMRA3	TEMRA4	TEMRA5	TEMRA6	LGL030	LGL037	LGL141	LGL148	LGL018	LGL028
ELP4	4017,474498	1,289759437	0,009783	11,589864	11,513657	11,303899	11,343967	10,921588	11,134929	12,337	11,22	11,059	12,248	11,506	12,224
SLC9A6	3756,41347	1,286559826	0,013602	11,286117	11,505394	10,913071	10,283316	11,35004	11,53707	12,68	12,102	12,553	11,118	12,322	11,532
PAM	9814,890099	1,246562746	0,019967	11,419991	13,126481	12,278737	12,822367	12,630714	12,791792	13,302	13,072	13,329	13,997	12,661	13,65
YIPF4	3222,584243	1,229501043	0,010308	10,525919	11,167068	10,062176	11,217812	11,610122	11,039928	11,68	12,21	11,5	11,865	10,955	12,035
ZNF655	11592,15924	1,199845189	0,014122	12,864665	12,027609	13,098365	12,948125	12,905652	13,217713	13,857	12,948	14,434	13,263	14,066	13,488
SLC25A53	1426,423863	1,158464084	0,030854	8,716657	10,502564	9,7606353	9,5693245	10,136792	9,9040836	11,131	10,186	10,147	10,264	10,312	10,762
FRYL	9152,112879	1,152390945	0,007983	11,937775	12,756547	12,146914	12,397317	12,871985	13,055569	12,932	13,241	12,617	13,384	12,902	13,528
ASXL2	3413,528719	1,148959457	0,015401	11,709337	11,250108	11,34519	10,826123	10,50157	10,95707	11,894	11,524	11,046	11,987	11,139	12,199
ARHGEF3	27275,51953	1,134008581	0,005764	13,781961	14,920088	13,896806	14,317135	14,073614	13,555351	14,367	14,979	15,246	14,939	14,775	14,903
NCALD	16257,36147	1,128550183	0,049546	11,94458	13,829404	13,603275	13,200904	13,390567	13,690499	14,076	13,879	13,491	14,199	13,497	14,638
TOX	16491,10554	1,110327597	0,038945	12,47729	14,042523	12,979747	13,878003	13,249989	13,286101	13,738	14,621	13,946	14,133	13,15	14,511
PRKG1	4801,544464	1,094099619	0,045517	11,779384	11,642568	11,135183	12,336559	11,188442	11,42255	12,993	12,085	12,12	12,166	12,45	11,844
UBA6	5691,808366	1,079858775	0,018917	11,09832	12,144366	11,964246	12,03185	11,338657	12,500986	12,741	12,365	12,971	12,803	12,983	12,654
RBM25	14385,04859	1,064887187	0,004031	13,144191	13,364644	13,566121	13,232507	13,143882	13,478621	13,929	13,347	14,545	14,006	14,388	13,764
CLOCK	1431,484863	1,035698319	0,021572	10,102241	10,142373	9,6881689	10,248273	9,8433955	9,7226774	10,937	9,8446	10,026	10,987	10,142	11,189
DYNC1LI2	4315,040273	1,034744704	0,017822	10,619928	11,788877	11,607411	11,472997	11,370869	12,085991	12,579	11,854	12,679	12,099	12,395	11,968
HS2ST1	2251,502344	1,015826654	0,04741	10,810415	10,622074	9,4748967	10,466511	11,018144	10,815612	11,87	11,236	10,798	11,029	10,856	10,915
TMCC1	2757,709534	1,014717059	0,049626	10,803124	11,321227	9,9628978	11,33442	10,559853	10,9701	11,458	10,682	11,153	11,58	11,414	11,462
LRBA	22884,26182	1,005169182	0,005786	13,65892	14,070829	14,460976	14,091245	13,631575	13,934344	14,983	14,305	14,907	14,665	14,9	14,363
RLF	16637,31371	-1,000332293	0,048057	13,272287	14,496358	14,399542	14,262575	14,564629	14,264993	14,098	13,292	14,293	13,604	14,171	13,151
DYRK2	9709,33783	-1,01183062	6,08E-05	13,263767	13,765113	13,634888	13,374663	13,890453	13,14026	12,929	13,092	13,014	12,676	12,747	13,003
MTMR10	4478,51595	-1,021548108	0,020165	13,080193	12,574509	12,419864	11,847673	11,881826	12,063788	12,073	11,914	12,247	11,517	12,027	11,349
NUFIP2	6163,983051	-1,022908525	0,005822	13,116621	12,573955	12,224659	13,121019	12,878503	13,070105	12,511	12,139	12,82	11,878	12,435	11,951
NR1D2	18714,66099	-1,028319715	0,006124	13,941648	14,621507	13,917786	14,68808	14,871066	14,485689	13,849	13,592	14,299	13,834	14,277	13,731
UBE2J1	15248,36636	-1,028845142	0,004598	14,214737	13,715727	14,181063	14,257834	14,575347	13,992847	13,381	13,796	14,082	13,012	13,822	13,166
AKIRIN1	12959,45586	-1,035210204	0,001241	14,19731	13,734117	13,396537	14,19506	14,166172	13,839772	13,339	13,602	13,578	13,104	13,555	12,993
FAM49A	8104,529001	-1,041668359	0,047679	14,053072	12,503076	13,315622	12,254639	12,872839	13,447418	12,115	12,897	13,058	12,646	11,801	12,38
ETV6	6864,575362	-1,048889303	0,048448	13,578994	12,784091	13,274933	12,194609	12,090484	13,278191	12,371	12,354	12,098	11,728	12,2	12,436
ARF6	42257,97311	-1,048980427	8,51E-06	15,791945	15,318566	15,657657	15,83169	15,812621	15,574871	14,869	15,309	15,183	14,754	15,056	14,932

ID	baseMean	log2FoldChange	padj	TEMRA1	TEMRA2	TEMRA3	TEMRA4	TEMRA5	TEMRA6	LGL030	LGL037	LGL141	LGL148	LGL018	LGL028
SF1	21697,35986	-1,054109732	0,000599	14,178032	14,940081	14,563055	14,878127	14,916836	14,594071	14,12	14,123	14,363	14,118	14,373	13,843
ABHD13	5175,029066	-1,062279194	1,69E-05	12,36787	12,497376	12,695644	12,594018	13,078184	12,514417	11,795	11,979	12,359	11,97	12,194	11,91
ZNF394	20198,5937	-1,0655329	0,014083	14,131051	14,50659	14,58697	14,823999	15,016059	14,191087	14,1	13,619	14,435	14,073	14,512	13,557
CHMP1B	8248,80708	-1,086746887	0,00044	13,633626	13,026783	13,122267	13,486728	13,240781	13,301747	12,501	13,205	12,509	12,128	12,509	12,885
ZDHHC3	7299,08095	-1,087271081	8,20E-05	13,370151	12,930982	13,226224	13,256292	13,200744	12,81305	12,581	12,881	12,27	12,136	12,53	12,27
PRKCD	6020,242369	-1,100316023	0,016419	13,807863	11,880064	12,241795	12,251193	12,801973	12,864212	11,89	12,076	12,226	12,2	12,356	12,162
TXNDC12	8184,286865	-1,112940257	0,000403	13,586644	12,869638	13,473443	13,353762	13,213727	13,263362	12,493	12,507	12,043	12,581	12,437	12,538
DUSP5	3655,504465	-1,122089227	0,025557	11,982084	10,761211	12,42408	12,539771	12,295881	11,968269	11,443	11,764	11,364	11,731	11,66	10,685
TMEM127	11179,97203	-1,129381254	0,009312	14,165762	13,359861	13,095785	14,142059	13,59123	13,71784	12,637	13,612	12,8	12,679	12,978	12,865
TFAM	3971,001764	-1,131814534	0,013455	12,087601	12,019558	12,410393	12,420332	12,532633	11,916765	11,349	11,717	12,138	10,959	12,068	11,279
MED26	3985,50829	-1,13298866	0,048057	12,27049	12,824141	11,397542	11,872108	12,314822	12,213376	12,05	11,074	12,111	11,299	12,196	11,006
TADA2B	10208,67201	-1,161263416	0,003253	13,134319	13,759823	13,19376	13,847864	14,075879	13,445239	13,008	13,247	12,495	12,542	12,529	12,754
EVI2A	2570,2675	-1,169138054	0,00362	11,545993	12,08245	11,436607	11,586318	11,525783	11,558746	10,968	10,785	10,413	10,225	10,911	11,374
NRAS	9733,247332	-1,170311124	0,017846	13,879319	12,785968	13,356444	13,389648	14,069212	13,340872	12,622	13,43	13,129	12,422	12,721	11,886
NKRF	2306,142513	-1,170886731	0,039651	10,563051	11,890609	12,007076	11,091034	11,713581	10,42632	11,202	10,92	11,255	10,515	10,946	10,375
MOSPD1	1345,471183	-1,173199362	0,012904	10,448358	10,582577	10,508602	9,4279966	11,439551	10,851756	9,739	9,8475	9,3282	9,7861	10,399	10,13
TSC22D2	9090,908131	-1,17755803	0,000338	13,133419	13,156751	13,822148	13,671865	13,431285	13,486623	12,986	12,961	12,56	13,014	13,059	12,633
KSR1	3131,860808	-1,181139123	0,015324	11,942559	11,597067	12,284191	10,958473	11,063527	12,627104	11,551	10,418	11,162	11,338	11,41	10,964
PGRMC2	2856,566377	-1,208515835	0,000144	11,718719	11,588831	11,259435	12,203874	12,306371	11,39214	11,245	11,019	11,087	10,796	11,354	10,81
ZADH2	2794,065674	-1,212820299	0,020416	11,341826	11,835537	12,734643	11,186172	11,454308	10,643008	10,312	11,106	10,848	10,732	10,686	11,053
ZDHHC7	9967,620647	-1,229867338	0,001431	13,550708	13,577427	12,739158	13,611057	13,759916	14,045188	13,134	12,518	13,414	12,638	12,799	12,479
DDIT4	25542,43704	-1,269067539	0,018052	15,466475	14,437922	15,121467	14,822923	14,889844	14,738073	14,328	14,522	13,942	12,663	14,732	13,939
ZFP36L1	9410,840273	-1,287831646	1,09E-05	14,033771	13,237069	13,488163	13,101213	13,319374	13,842416	12,821	13,162	12,526	12,615	12,66	12,652
PAPD5	7517,308382	-1,29274882	0,00999	12,825179	12,754598	13,685917	13,40287	13,126954	13,100883	13,012	11,703	12,669	12,117	13,104	11,684
YTHDF3	14004,92312	-1,298738948	0,002965	14,138979	13,311218	13,406143	14,311237	14,560456	14,328685	13,272	13,65	13,86	12,973	13,537	12,77
PNRC1	83599,54414	-1,31568593	3,79E-07	16,73818	16,584905	16,843376	16,915626	16,772176	16,361924	15,778	16,096	15,828	15,898	16,18	15,842
THRA	2015,533162	-1,323701448	0,016327	10,746785	11,836774	10,467636	11,009832	11,477244	11,586884	10,5	9,8646	9,8899	11,32	9,6697	10,024
MSL2	11457,67851	-1,342780798	3,46E-05	13,962143	14,065453	13,352513	14,199357	13,846444	13,356325	12,998	13,354	13,275	12,577	13,21	12,892
S1PR1	7860,386638	-1,415055695	0,046265	12,357552	13,519386	12,945759	13,6441	13,641937	12,657215	12,368	11,715	10,817	11,296	11,842	12,85

ID	baseMean	log2FoldChange	padj	TEMRA1	TEMRA2	TEMRA3	TEMRA4	TEMRA5	TEMRA6	LGL030	LGL037	LGL141	LGL148	LGL018	LGL028
HMBS	1286,169807	-1,453185572	0,007622	10,732414	10,86985	11,506018	9,2630769	10,437544	9,7609138	9,6272	9,5408	8,9209	9,9421	9,9238	9,7079
KLHL15	2566,809905	-1,46559848	0,024886	11,080891	10,227185	12,368285	11,527328	11,666111	11,864885	11,234	10,538	11,588	10,762	10,917	10,186
SMG7	10706,8603	-1,488634132	0,005031	14,861326	12,921371	13,109485	13,200726	12,673662	13,853639	12,854	12,604	13,119	13,164	12,872	12,554
SMAD7	9124,457953	-1,517454816	0,000624	13,329835	13,870797	13,466055	13,733961	13,609067	12,962943	12,83	12,939	13,031	12,291	12,391	12,631
PURB	3770,900197	-1,538119626	4,27E-05	12,295089	12,097177	11,864221	12,495456	12,540772	12,199553	11,352	11,426	11,697	10,922	11,418	10,515
DNAJB1	37653,48913	-1,538280653	0,000438	15,331356	15,138969	15,618244	16,2477	15,570686	15,128356	14,691	15,129	14,167	13,696	14,905	14,459
BAG4	4029,489566	-1,540663087	0,000122	11,942977	12,313087	12,422047	12,125683	12,654554	12,559709	11,266	11,286	12,116	11,398	11,851	10,819
SPTY2D1	6531,709448	-1,55315276	3,71E-07	12,788622	12,744512	13,071804	13,202674	13,523584	12,899506	11,817	12,291	12,325	12,218	12,582	11,827
BCL6	2754,666356	-1,553632109	0,004847	12,307871	11,575745	11,139848	11,92534	11,438251	11,924356	11,1	10,633	11,494	10,615	11,561	9,8232
SH2B3	10529,21849	-1,558424195	0,000409	14,050485	13,374184	12,87395	13,302921	13,645717	14,447074	13,195	12,752	12,837	12,974	13,116	12,385
ZFAND5	16608,90472	-1,56362244	3,92E-07	14,85294	14,163941	14,209333	14,440374	14,316814	14,413549	13,637	13,516	13,646	13,614	13,83	13,354
FAM46C	13396,26226	-1,569930029	0,004028	14,052025	12,555416	14,412488	14,651614	14,061562	13,632427	13,288	13,41	13,06	12,534	13,755	12,528
BHLHE40	19151,4544	-1,597183723	0,000948	14,999524	13,687416	14,557243	14,776661	14,953285	14,069978	13,847	13,661	13,83	13,152	14,26	13,187
HNRNPA0	19319,48653	-1,631214938	2,29E-08	14,531781	14,625513	14,538701	15,047545	14,688761	14,356378	13,719	13,819	13,927	13,408	13,99	13,307
SLC25A37	2291,710674	-1,65449558	0,038532	12,592164	11,518002	9,938647	9,3873274	10,487617	11,706495	10,636	9,9005	10,456	8,9684	10,384	10,473
FKBP1A	1601,271819	-1,65456701	1,08E-05	11,420471	10,981372	10,799479	11,24359	10,673721	11,031935	9,8096	10,241	9,3065	9,9225	9,4983	10,229
SERTAD2	3992,568771	-1,711107298	4,64E-06	12,730265	11,695858	12,443315	11,584702	12,409287	12,820634	11,364	11,07	11,572	11,148	11,284	11,465
BTG2	18518,67109	-1,718658654	1,76E-07	14,966042	14,316881	14,410749	14,66211	14,442248	14,666843	13,598	14,11	13,177	13,225	13,235	13,222
RASSF2	3400,65624	-1,81183807	0,025458	12,880496	10,347249	12,028468	11,598825	11,826102	12,313694	10,115	9,0772	10,18	10,655	10,392	11,107
CBX4	17655,77943	-1,868915264	0,000359	13,812651	14,296864	14,269	14,938742	15,108746	14,037837	13,687	13,552	13,81	12,962	14,041	12,841
SLC2A3	31129,39226	-1,88064899	2,07E-05	15,629255	14,708027	15,429465	15,787444	15,105902	15,094938	14,272	14,449	14,203	13,504	14,979	13,797
APLP2	19051,46439	-2,234135511	2,81E-05	15,490154	13,584691	14,163781	14,419856	14,398178	14,898354	13,27	13,036	12,243	13,205	12,775	13,491
NR4A3	2437,210632	-2,41293588	0,002421	12,054579	10,889228	11,056976	12,307055	11,268075	11,540582	10,703	9,4278	10,429	9,6075	11,12	9,4435
PLXDC2	4657,31288	-2,507205805	0,027445	13,659122	10,284222	12,182606	8,8539913	10,982854	13,032848	11,157	8,7841	10,226	10,45	11,345	10,463
SOCS3	8357,373786	-2,624068071	4,92E-05	14,518277	12,543493	12,397742	12,845188	12,501146	13,947124	12,353	12,29	11,468	12,121	11,715	11,052
DUSP6	1990,34687	-2,686818501	2,65E-08	12,496102	11,148761	10,964622	10,846725	10,913173	11,110262	9,7901	9,7054	9,1658	9,4571	10,358	9,9145
PSAP	156735,7589	-2,79833216	4,40E-10	18,683189	16,737746	17,198545	16,880805	17,231139	18,208837	15,808	16,223	15,73	16,08	15,944	15,787
SLC30A1	3771,754063	-2,888055482	1,31E-10	12,711404	11,394519	12,326693	12,520549	12,187334	12,647043	11,277	10,4	10,513	10,333	10,276	10,397
SNN	2689,578334	-3,293835934	2,09E-07	12,475008	10,570396	12,051217	11,088924	11,274077	12,3666	10,249	10,228	10,134	8,6352	9,3763	10,114

ID	baseMean	log2FoldChange	padj	TEMRA1	TEMRA2	TEMRA3	TEMRA4	TEMRA5	TEMRA6	LGL030	LGL037	LGL141	LGL148	LGL018	LGL028
CKAP4	2772,22495	-3,363419251	4,28E-07	12,64652	11,438135	11,692472	9,8170707	10,711575	12,585256	9,9981	8,7559	9,1433	10,004	9,7657	8,808
IRS2	2228,871742	-3,411355214	3,13E-05	12,022536	11,257622	11,89263	10,521104	11,59803	11,359237	9,7187	8,0724	8,0913	8,1885	9,8226	10,046
VCAN	10454,42756	-4,491014914	1,86E-07	15,265513	12,0114	12,257066	10,711087	12,196324	14,653897	11,744	10,529	10,231	10,393	10,777	10,379

Supplementary table 5 Differentially expressed miR-181a mRNA targets between T-LGL cells and healthy control TEMRA CD8 Lymphocytes

Supplementary table 6. Primers used to generate miR181ab pLX301 vector

Primer name	Sequence (5' -> 3')	Application
miR181a/b_FW2	CAATGTGATGTGGAGGTTTG	Amplification of miR181 cluster from gDNA KG-1 cell line
miR181a/b_R2	ACAACAGTAGGAAGGTGAG	Amplification of miR181 cluster from gDNA KG-1 cell line
MSCV-SV40-FW	TGTGGAATGTATATCAGTTAG	Amplification of SV40 promoter from pBABE-puro(Plasmid #1764, addgene)
pBABE-SV40-RV	CTCGGTCATGGTAAGCTTTTTG	Amplification of SV40 promoter from pBABE-puro(Plasmid #1764, addgene)<br>Underscore corresponds with SV40_miR181a/b_FW and pB_SV40_mir141_200c_FW
SV40_miR181a/b_FW	**GGCTTTTGCAAAAAGCTTACCATGACCGAG**CAATGTGATGTGGAGGTTTG	Adding a 30 nt overhang (bold) to miR181 cluster to link SV40 promoter to miR181 cluster.<br>Underscore corresprونds to pBABE-SV40-RV
MSCV-SV40-NheI-FW	**GCTAGC**TGTGGAATGTGTGTCAGTTAG	Linking SV40 promoter to miR141 cluster and miR181 cluster.<br>Adding a *NheI* restriction site (bold) to 5’-end of SV40-miR141 cluster and SV40-miR181 cluster
miR181a/b_AgeI_RV2	**ACCGGT**ACAACAGTAGGAAGGTGAG	Adding a *AgeI* restriction site (bold) to 3’-end of SV40-miR181ab insert.
